# Proactive integrated consultation-liaison psychiatry and time spent in hospital by older medical inpatients in England (The HOME Study): a multicentre, parallel-group, randomised controlled trial

**DOI:** 10.1016/S2215-0366(24)00188-3

**Published:** 2024-09

**Authors:** Michael Sharpe, Jane Walker, Maike van Niekerk, Mark Toynbee, Nicholas Magill, Chris Frost, Ian R White, Simon Walker, Ana Duarte, Colm Owens, Chris Dickens, Annabel Price, HOME Study Team, Michael Sharpe, Jane Walker, Maike van Niekerk, Mark Toynbee, Nicholas Magill, Chris Frost, Ian R White, Simon Walker, Ana Duarte, Colm Owens, Chris Dickens, Annabel Price, Peter Aitken, Tomasz Bajorek, Gunes Berk, Rhian Bold, Katy Burke, Jonathan Burns, Shelley Campbell, Hannah Chaitow, Felix Clay, Michael Daly, Tobit Emmens, Elliot Hampsey, Naomi Hannaway, Jessica Harris, Rowan Harwood, Laura Hill, Harriet Hobbs, Laura Hollands, Sophie Howitt, Rhian Kant, Sarah E Lamb, Daniel Lasserson, Hochang Benjamin Lee, Eleanor Macey, Aelfrida Palmer, Julie Philps, Louise Pollard, Isabelle Rocroi, Anna Scholz, Sasha Shepperd, Gabrielle Sirois-Giguere, Luke Solomons, Ben Steward, Will Turner, Michael Yousif

**Affiliations:** a Psychological Medicine Research, University of Oxford Department of Psychiatry, Warneford Hospital, Oxford, UK; b Department of Medical Statistics, London School of Hygiene and Tropical Medicine, London, UK; c MRC Clinical Trials Unit at UCL, London, UK; d Centre for Health Economics, University of York, York, UK; e NHS Devon Mental Health, Learning Disability and Neurodiversity Provider Collaborative, Devon, UK; f University of Exeter Medical School, University of Exeter, Exeter, UK; g Cambridgeshire and Peterborough NHS Foundation Trust, Cambridge, UK

## Abstract

**Background:**

Older people admitted to hospital in an emergency often have prolonged inpatient stays that worsen their outcomes, increase health-care costs, and reduce bed availability. Growing evidence suggests that the biopsychosocial complexity of their problems, which include cognitive impairment, depression, anxiety, multiple medical illnesses, and care needs resulting from functional dependency, prolongs hospital stays by making medical treatment less efficient and the planning of post-discharge care more difficult. We aimed to assess the effects of enhancing older inpatients’ care with Proactive Integrated Consultation-Liaison Psychiatry (PICLP) in The HOME Study. We have previously described the benefits of PICLP reported by patients and clinicians. In this Article, we report the effectiveness and cost-effectiveness of PICLP-enhanced care, compared with usual care alone, in reducing time in hospital.

**Methods:**

We did a parallel-group, multicentre, randomised controlled trial in 24 medical wards of three English acute general hospitals. Patients were eligible to take part if they were 65 years or older, had been admitted in an emergency, and were expected to remain in hospital for at least 2 days from the time of enrolment. Participants were randomly allocated to PICLP or usual care in a 1:1 ratio by a database software algorithm that used stratification by hospital, sex, and age, and randomly selected block sizes to ensure allocation concealment. PICLP clinicians (consultation-liaison psychiatrists supported by assisting clinicians) made proactive biopsychosocial assessments of patients’ problems, then delivered discharge-focused care as integrated members of ward teams. The primary outcome was time spent as an inpatient (during the index admission and any emergency readmissions) in the 30 days post-randomisation. Secondary outcomes were the rate of discharge from hospital for the total length of the index admission; discharge destination; the length of the index admission after random allocation truncated at 30 days; the number of emergency readmissions to hospital, the number of days spent as an inpatient in an acute general hospital, and the rate of death in the year after random allocation; the patient's experience of the hospital stay; their view on the length of the hospital stay; anxiety (Generalized Anxiety Disorder-2); depression (Patient Health Questionnaire-2); cognitive function (Montreal Cognitive Assessment-Telephone version); independent functioning (Barthel Index of Activities of Daily Living); health-related quality of life (five-level EuroQol five-dimension questionnaire); and overall quality of life. Statisticians and data collectors were masked to treatment allocation; participants and ward staff could not be. Analyses were intention-to-treat. The trial had a patient and public involvement panel and was registered with ISRTCN (ISRCTN86120296).

**Findings:**

2744 participants (1399 [51·0%] male and 1345 [49·0%] female) were enrolled between May 2, 2018, and March 5, 2020; 1373 were allocated to PICLP and 1371 to usual care. Participants’ mean age was 82·3 years (SD 8·2) and 2565 (93·5%) participants were White. The mean time spent in hospital in the 30 days post-randomisation (analysed for 2710 [98·8%] participants) was 11·37 days (SD 8·74) with PICLP and 11·85 days (SD 9·00) with usual care; adjusted mean difference –0·45 (95% CI –1·11 to 0·21; p=0·18). The only statistically and clinically significant difference in secondary outcomes was the rate of discharge, which was 8.5% higher (rate ratio 1·09 [95% CI 1·00 to 1·17]; p=0·042) with PICLP—a difference most apparent in patients who stayed for more than 2 weeks. Compared with usual care, PICLP was estimated to be modestly cost-saving and cost-effective over 1 and 3, but not 12, months. No intervention-related serious adverse events occurred.

**Interpretation:**

This is the first randomised controlled trial of PICLP. PICLP is experienced by older medical inpatients and ward staff as enhancing medical care. It is also likely to be cost-saving in the short-term. Although the trial does not provide strong evidence that PICLP reduces time in hospital, it does support and inform its future development and evaluation.

**Funding:**

UK National Institute for Health and Care Research.

**Research in context**

**Evidence before this study**

The evidence before our study is described in two published reviews. A systematic meta-review (Siddique et al, 2021) summarised the evidence for all interventions designed to reduce time in hospital for older medical inpatients, and concluded that none were consistently effective. Another systematic review (Oldham et al, 2019) summarised the evidence for the effectiveness of proactive and integrated consultation-liaison psychiatry service models. It used searches of four databases up to May, 2019 (PubMed, Embase, PsycINFO, and Cochrane Library) with the following terms ((“psychiatry” OR “psychiatr*”) AND (“consult” OR “consultat*” OR “liaison”) OR (“psychosomatic”)) AND (“proactive” OR “screen*” OR “early” OR “rapid” OR “prompt”) AND (“length” OR “cost” OR “quality”). It found evidence from non-randomised studies that these service models might reduce time in hospital for working-age adults.

**Added value of this study**

To the best of our knowledge, The HOME Study is the first randomised controlled trial of Proactive Integrated Consultation-Liaison Psychiatry (PICLP), and the first study of its effectiveness in older medical inpatients. Its findings indicate that PICLP can be delivered at scale, is seen by older inpatients and ward staff as enhancing medical care, and might be cost saving in the short-term. However, the trial findings do not provide sufficient evidence to recommend the implementation of PICLP to reduce time in hospital for older medical inpatients.

**Implications of all the available evidence**

We still do not have evidence from randomised controlled trials that any intervention is consistently effective in reducing the time older patients spend in hospital. Consequently, new approaches are still needed. PICLP shows promise in this regard, and our findings suggest ways in which it could be further developed. These include more intensive delivery, a sharper focus on patients at very high risk of longer stays, and greater influence on both inpatient and out-of-hospital care.

## Introduction

Older people (≥65 years) who are admitted to acute medical wards in an emergency often remain there for long periods.^1^ Prolonged inpatient stays are bad for older people because they increase their risk of hospital-acquired illnesses, mental and physical deterioration, and loss of independence after discharge.^1^, ^2^, ^3^ They are also bad for health services because they increase the cost of care and reduce the availability of beds for new admissions.^1^ Although the problem of prolonged stays is set to worsen as the number of older people in the population increases, an effective solution remains elusive.^4^

Growing evidence suggests that the complexity of older patients’ problems is crucial in prolonging hospital stays. Older patients typically have psychiatric and psychosocial problems, including cognitive impairment, depression, and anxiety, as well as multiple medical illnesses and care needs resulting from functional dependency.^5^, ^6^, ^7^, ^8^, ^9^ This biopsychosocial complexity leads to less efficient medical treatment, difficulty arranging post-discharge care, and consequently, prolonged hospital stays.^10^, ^11^, ^12^, ^13^ Proactive Integrated Consultation-Liaison Psychiatry (PICLP) is a novel way of enhancing the care of patients in acute medical wards.^14^ PICLP was designed to help ward teams manage biopsychosocial complexity, and thereby reduce the time that older medical inpatients spend in hospital. PICLP was inspired by innovations in the care of medical inpatients in the USA.^15^, ^16^ In PICLP-enhanced medical care, consultation-liaison psychiatrists and assisting clinicians make proactive and comprehensive biopsychosocial assessments of all older patients’ problems soon after their admission to the ward, and then deliver discharge-focused clinical care as integrated members of the ward teams.

The HOME Study was a randomised controlled trial, which aimed to study the effects of PICLP-enhanced care (hereafter referred to as PICLP) for older medical inpatients.^17^ We have previously described the positive experiences of PICLP reported by the clinicians who delivered it in the trial, staff of the medical wards in which it was provided, and patients who received it.^18^, ^19^ In this Article, we report on the effectiveness and cost-effectiveness of PICLP in reducing the time that older medical inpatients spend in hospital, compared with usual care.

## Methods

### Study design and participants

The HOME Study was a parallel, two-group, multicentre, randomised, controlled, superiority trial in 24 acute medical wards of three English acute general hospitals. The hospitals, which are all publicly funded National Health Service (NHS) teaching hospitals, each provide care for a geographically defined population. The trial was approved by the South-Central Research Ethics Committee (17/SC/0497) and the Confidentiality Advisory Group (17/CAG/0160). It was overseen by a Trial Steering Committee, a Data Monitoring Committee, and a Patient and Public Involvement Panel (appendix p 3). The patient and public involvement panel members all had experience of being an older medical inpatient or a family member of an older patient, and members contributed to planning recruitment procedures, developing the PICLP service manual (appendix pp 26–39), choosing the outcome measures, training research staff, and interpreting the trial findings. The trial protocol and statistical analysis plan have been published.^17^, ^20^ The study is registered with the ISRCTN registry (ISRCTN86120296).

Panel **Proactive integrated consultation-liaison psychiatry: stages of delivery**

**Stage 1: the proactive biopsychosocial clinical assessment**

• The consultant psychiatrist interviews the patient soon after their admission to the medical ward, and the assisting clinician gathers information from the patient's family, ward team, and medical records
• The psychiatrist then makes a comprehensive list of the patient's problems, including any psychiatric diagnoses. They use this information to identify and prioritise the problems most likely to increase the time the patient will spend in hospital

**Stage 2: formulation and communication of the discharge-focused action plan**

• The psychiatrist formulates an action plan designed to address the prioritised problems
• They discuss the action plan with the patient, their family, and the ward team, and agree how it will be implemented

**Stage 3: integrated implementation of the discharge-focused action plan**

• The psychiatrist and assisting clinicians work with the ward team to implement the action plan and deliver interventions, including:
  • Enabling the ward team to provide biopsychosocial care
  • Collaborating with the patient, ward team, family, primary care, and social care to plan effective discharge
  • Giving specific advice on the management of psychiatric disorders and other psychological problems (eg, the diagnosis and treatment of depression) and the use of medications (eg, when to prescribe drugs for symptoms of dementia)
  • Discussing the need for, and timing of, medical investigations that would prolong the patient's hospital stay (eg, suggesting the deferral of a non-urgent scan until after discharge)
  • Providing psychological interventions directly to the patient (eg, doing graded exposure therapy to help the patient to overcome anxiety about rehabilitation after a fall)
  • Helping the patient's family and other carers to accurately anticipate the patient's needs after discharge (eg, by explaining the difference between transient delirium and progressive dementia)
• The assisting clinicians monitor the patient's progress daily. They review and establish which problems are currently impeding the patient's discharge so that the action plan can be updated as needed

**Stage 4: communication with out-of-hospital care providers at the time of discharge**

• The psychiatrist and assisting clinicians communicate with out-of-hospital care providers about unresolved problems and make recommendations for further care, including:
  • Specific medical advice to the patient's primary care provider
  • Referral to a community psychiatric service
  • Advice to social care providers

We identified potential trial participants by screening all consecutive admissions to the acute medical wards for eligibility. Patients were eligible if they were aged at least 65 years; had been admitted in an emergency; and were expected to remain in hospital for at least 2 days from the time of enrolment. We excluded patients if they had already been in hospital for 7 days or more; their clinicians predicted that they would die before discharge; they had already been referred to the traditional consultation-liaison psychiatry service; they were unable to read or speak English; they had already been enrolled during a previous admission; or their participation was considered to be clinically or practically inappropriate.

We determined whether patients were eligible to participate using information from medical records and ward clinicians. We obtained written informed consent from those who had capacity. If a patient lacked capacity, we identified a consultee who could advise on whether they should be enrolled in the trial, in accordance with the Mental Capacity Act 2005, England and Wales.^21^

### Randomisation and masking

Participants were individually randomly allocated to either PICLP or usual care in a 1:1 ratio by a database software algorithm (program written by CF and implemented with a changed seed by the Oxford Clinical Trials Unit). Individual randomisation (rather than cluster randomisation of medical wards) was used because PICLP was designed to affect care predominantly at the patient level, and because natural clusters do not exist as patients and staff move between wards. The randomisation sequence was stratified by hospital, sex, and age (65–74 years, 75–84 years, and ≥85 years), and used randomly selected block sizes to ensure allocation concealment. Researchers accessed the database via a secure website and only received the participant's treatment allocation after entering key baseline data. Staff who collected outcome data and trial statisticians were masked to allocated interventions. Participants and ward staff could not be masked to treatment allocation due to the nature of PICLP.

### Procedures

PICLP has been described in detail in a separate publication.^14^ PICLP is delivered by consultant (attending) consultation-liaison psychiatrists, supported by assisting clinicians (doctors or allied health professionals). The PICLP clinicians proactively assess patients soon after their admission to the ward. They see every older medical inpatient, as nearly all will have complex biopsychosocial problems. The assessment informs their work as integrated members of the ward team, co-managing the patients’ care and discharge planning. As well as directly contributing to patients’ care with pharmacological and psychological interventions, the PICLP clinicians work systemically with families, ward team members, and out-of-hospital care providers. To ensure consistency, the PICLP service model is operationalised in a service manual and a clinicians’ workbook with checklists (appendix pp 40–48). The manual specifies four stages of PICLP delivery, as described in the panel. The PICLP service model differs substantially from traditional consultation-liaison psychiatry practice, in which psychiatrists see the small proportion of patients referred to them and only provide advice on their care.^22^

In The HOME Study, PICLP started soon after random allocation and continued for a maximum of 30 days. It was delivered by a total of seven consultation-liaison psychiatrists and eight assisting clinicians (appendix p 3), working in three hospital-based teams. The psychiatrists each had at least 5 years of clinical experience after specialist training. Six of the assisting clinicians were psychiatrists in training and two were experienced psychiatric occupational therapists. The PICLP clinicians were all trained to provide care according to the service manual. Their training has been described in detail in a previous publication.^18^ It comprised workshops and the practice of aspects of the service model in their own hospitals. Each clinician's adherence to the manual was assessed by observation of their practice in the clinical setting. After completing training, the clinicians met weekly for peer supervision by videoconferencing across the three hospitals. Adherence to the service manual was monitored by repeated direct observation of the PICLP clinicians’ work and review of every patient's completed workbook.

Usual care was provided by the ward teams and was not specified or restricted in the trial to ensure that it represented current practice. The hospitals each had a traditional consultation-liaison psychiatry service to which patients could be referred.

### Outcomes

The primary outcome was the number of days spent as an inpatient (during the index admission and any emergency readmissions to acute general hospitals) in the 30 days post-randomisation. This period was chosen because patients are usually discharged within 14 days, and 30 days would therefore capture both the index admission and any early readmissions. The secondary outcomes were the rate of discharge from hospital (discharges per day) for the total length of the index admission; discharge destination for participants who had been admitted from a private residence; the length of the index admission after random allocation truncated at 30 days, as recommended by the Trial Steering Committee after publication of the statistical analysis plan; the number of emergency readmissions to hospital in the year after random allocation; the number of days spent as an inpatient in an acute general hospital in the year after random allocation; and the rate of death in the year after random allocation. We collected these data from the participants’ medical records, the NHS Hospital Episode Statistics database, and the Office for National Statistics civil registration database. Additional secondary outcomes were the patient's experience of the hospital stay (0–10 scale from terrible to excellent); the patient's view on the length of the hospital stay (too short, about right, or too long); the patient's anxiety (Generalized Anxiety Disorder-2 [GAD-2]);^23^ depression (Patient Health Questionnaire-2 [PHQ-2]);^23^ cognitive function (Montreal Cognitive Assessment-Telephone version [MoCA-T]);^24^ independent functioning (Barthel Index of Activities of Daily Living);^25^, ^26^ health-related quality of life (five-level EuroQol five-dimension questionnaire [EQ-5D-5L]);^27^ and overall quality of life (0–10 scale). We collected data from participants using brief face-to-face interviews at baseline (the data were not made available to the PICLP clinicians or medical ward staff) and using telephone interviews (supplemented by in-person visits when necessary) at 1 month and 3 months post-random allocation. When possible, we collected these data from participants themselves. If a participant was unable to provide data, even with help, we asked a proxy (the consultee, or another family member, friend, or clinician) to provide data on their behalf.

We defined serious adverse events as deaths from any cause in the 30 days post-randomisation.

### Statistical analysis

Using pre-trial data from the three hospitals, we estimated that two trial groups of 1794 participants each would give 90% statistical power (and two groups with 1340 participants each would give 80% statistical power) at the 5% significance level (two-sided test) to detect a difference of at least 1 day (from 9 days to 8 days; SD 9 days) in the mean number of days spent in hospital in the 30 days post-randomisation, allowing for 5% loss to follow-up. We sought to detect a difference of 1 day, as there is no agreed minimally important difference on this measure and this amount of time was considered to be meaningful by both patients and clinicians.

All analyses were conducted by original assigned groups (intention-to-treat). The effect of PICLP on each outcome is a weighted mean of the three hospital-specific treatment effects, weighted according to number of participants per hospital. We analysed the primary outcome using linear regression. We did not do a per-protocol analysis because 99% of participants allocated to PICLP received it (defined as having had at least stages 1 and 2 of PICLP delivery; appendix p 7). We used Cox models to compare rates of discharge (censoring deaths) and rates of death (censored at time of last contact for participants whose mortality status was missing) in the year after random allocation, and Poisson regression with robust standard errors for number of emergency readmissions. The remaining secondary outcomes were analysed using linear and logistic regression. We included the stratification variables, medical ward, and hospital-by-treatment interaction terms as fixed effects in all our models. Anxiety, depression, cognitive function, independent functioning, health-related quality of life, and overall quality of life were also adjusted for corresponding baseline scores. For the primary outcome, we explored effects in subgroups by adding, one at a time, interactions between treatment and each of the stratification factors. We used multiple imputation, using chained equations with 100 replications, to handle missing patient-reported data. The imputation models included variables in the main model, values of the variable being analysed at other timepoints, and pre-defined auxiliary variables. We made no assumptions regarding patient-reported outcomes for those who had died, and therefore discarded any imputed values for deceased participants. We used a two-sided significance level of 0·05 throughout, with the only exception being the overall comparison of deaths, which used 0·045 to take account of interim analyses done by the Data Monitoring Committee. Statistical analyses were done using Stata version 17; full details are given in the published statistical analysis plan.^20^

The COVID-19 pandemic had a major effect on hospital admissions and on deaths of older patients from March, 2020. We therefore did sensitivity analyses (pre-specified after the statistical analysis plan was published and before the end of the trial) to address effects of the pandemic on the outcomes that were measured over the year post-randomisation. We split the follow-up period into before (and including) and after March 1, 2020, for number of emergency readmissions and number of days in hospital, and censored at this date for deaths (see appendix p 19 for details).

### Economic analysis

We evaluated the cost-effectiveness of PICLP, from the hospital perspective, over three time horizons (1 month, 3 months, and 12 months) using the aforementioned data on hospital admissions, deaths, and health-related quality of life. For each time horizon, we calculated the total cost of inpatient care (valued at 2020–21 prices and expressed in pounds sterling). This comprised the costs of PICLP delivery, the index admission, and subsequent emergency admissions to acute general hospitals. Health outcomes were expressed in quality-adjusted life-years (QALYs), which we calculated using data on deaths and EQ-5D-3L scores at baseline, 1 month, and 3 months (because no data were collected from participants at 12 months, EQ-5D-3L scores were assumed to remain constant after 3 months for participants who were alive beyond that timepoint). We estimated incremental cost-effectiveness ratios (incremental cost per QALY) and present the probabilities of PICLP being cost-saving and cost-effective at a range of cost-effectiveness thresholds commonly used in the UK health system.^28^

### Role of the funding source

The funder of the study had no role in study design, data collection, data analysis, data interpretation, or writing of the report.

## Results

**Figure 1:**
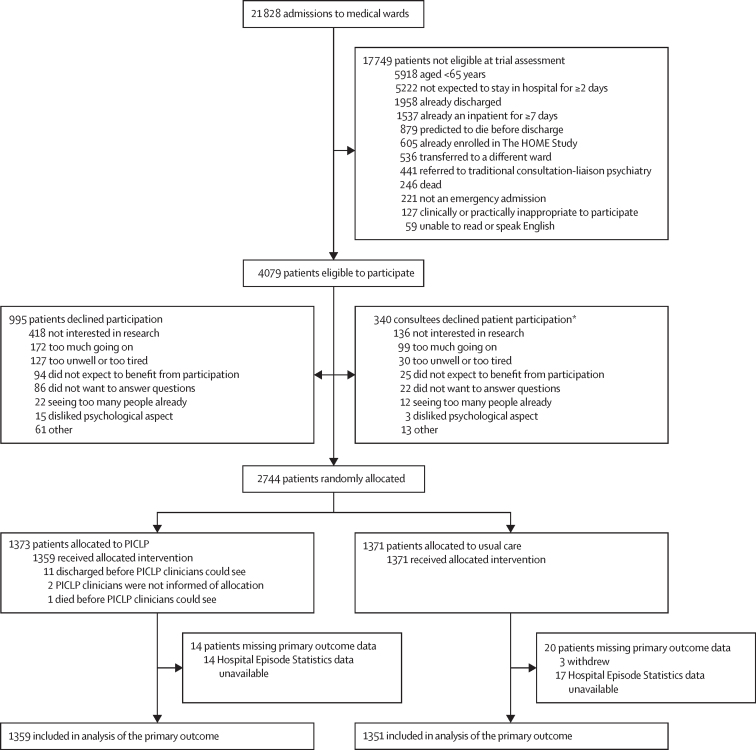
Trial profile PICLP=proactive integrated consultation-liaison psychiatry. *If a patient lacked capacity, we identified a consultee who could advise on whether they should be enrolled in the trial in accordance with the Mental Capacity Act 2005, England and Wales.

Between May 2, 2018, and March 5, 2020, when recruitment was terminated early due to the COVID-19 pandemic, there were 21 828 admissions to the acute medical wards (figure 1). Of the 4079 patients found to be eligible, 2744 (67·3%) were enrolled and randomly allocated (1623 [59·1%] gave informed consent and 1121 [40·9%] had a consultee agree to their participation; see appendix p 5). 1373 participants were allocated to PICLP and 1371 participants to usual care. We obtained data from medical records and databases, including the primary outcome data, for 2710 (98·8%) participants. We also collected data from 2105 participants (90·1% of 2332 alive) at 1 month and 1797 participants (85·0% of 2115 alive) at 3 months (see appendix p 16 for details).

Participants’ baseline characteristics are described in table 1. The mean age was 82·3 years (range 65–102; SD 8·2), 1399 (51·0%) of 2744 participants were male and 1345 (49·0%) were female, and 2565 (93·5%) participants were White. Most participants were cognitively impaired, a substantial proportion had clinically significant symptoms of anxiety and depression, and almost all had multimorbidity (table 1).

**Table 1:** The HOME Study participants' baseline characteristics

		**PICLP (N=1373)**	**Usual care (N=1371)**
Age, years		82·3 (8·2)	82·3 (8·2)
Age group			
	65–74 years	308 (22·4%)	307 (22·4%)
	75–84 years	484 (35·3%)	488 (35·6%)
	≥85 years	581 (42·3%)	576 (42·0%)
Sex			
	Female	673 (49·0%)	672 (49·0%)
	Male	700 (51·0%)	699 (51·0%)
Ethnic group			
	White or White British	1283 (93·4%)	1282 (93·5%)
	Other*	8 (0·6%)	9 (0·7%)
	Missing data	82 (6·0%)	80 (5·8%)
Married or has long-term partner		602 (43·8%)	615 (44·9%)
Retired or not working		1351 (98·4%)	1335 (97·4%)
Usual place of residence			
	Private residence	1251 (91·1%)	1245 (90·8%)
	Care home or nursing home	87 (6·3%)	95 (6·9%)
	Sheltered accommodation or assisted living	35 (2·5%)	31 (2·3%)
Lives alone		616 (44·9%)	598 (43·6%)
Hospital			
	Royal Devon and Exeter (Exeter)	637 (46·4%)	636 (46·4%)
	John Radcliffe (Oxford)	530 (38·6%)	528 (38·5%)
	Addenbrooke's (Cambridge)	206 (15·0%)	207 (15·1%)
Days in hospital pre-enrolment		3·5 (1·7)	3·4 (1·7)
Days in medical ward pre-enrolment		2·3 (1·5)	2·2 (1·5)
Number of emergency admissions in the past year, median (range)		1 (0–38)	0 (0–11)
Number of medical conditions recorded at hospital admission, median (range)		4 (0–20)	4 (0–20)
Multimorbidity (≥2 current medical conditions)		1250 (91·0%)	1242 (90·6%)
Number of medications prescribed at the time of enrolment, median (range)		10 (1–33)	10 (0–36)
Anxiety (GAD-2, 0–6)†			
	Mean score	2·5 (2·2)	2·6 (2·1)
	Score ≥3 (clinically significant symptoms)	587 (42·8%)	604 (43·9%)
	Missing data	32 (2·3%)	33 (2·4%)
Depression (PHQ-2, 0–6)†			
	Mean score	2·6 (2·1)	2·5 (2·1)
	Score ≥3 (clinically significant symptoms)	647 (47·1%)	629 (45·9%)
	Missing data	43 (3·1%)	32 (2·3%)
Cognitive function (MoCA-T, 0–30)‡§			
	Mean score	13·0 (8·4)	13·0 (8·6)
	Score 26–30 (normal cognitive function)	78 (5·7%)	95 (6·9%)
	Score 18–25 (mild cognitive impairment)	374 (27·2%)	344 (25·1%)
	Score 10–17 (moderate cognitive impairment)	377 (27·5%)	384 (28·0%)
	Score 0–9 (severe cognitive impairment)	409 (29·8%)	424 (30·9%)
	Missing data	135 (9·8%)	124 (9·0%)
Independent functioning (Barthel, 0–100)§			
	Mean score	46·6 (28·5)	45·1 (29·3)
	Score <40 (complete dependence on others)	564 (41·1%)	602 (43·9%)
	Missing data	0	1 (0·1%)
Health-related quality of life (EQ-5D-5L)§			
	Mean score	0·3 (0·4)	0·3 (0·4)
	Missing data	20 (1·5%)	18 (1·3%)
Overall quality of life (0–10)§			
	Mean score	5·0 (2·8)	4·9 (2·8)
	Missing data	21 (1·5%)	25 (1·8%)

Data are n (%) or mean (SD), unless otherwise specified. Barthel=Barthel Index of Activities of Daily Living. EQ-5D-5L=five-level EuroQol five-dimension questionnaire. GAD-2=Generalized Anxiety Disorder-2. MoCA-T=Montreal Cognitive Assessment-Telephone version. PHQ-2=Patient Health Questionnaire-2. PICLP=proactive integrated consultation-liaison psychiatry.

* For PICLP: Black or Black British (n=3); Asian or Asian British (n=2); Mixed (n=1); Any other background (n=2). For usual care: Black or Black British (n=1), Asian or Asian British (n=5); Mixed (n=3).

† Higher scores indicate worse health.

‡ MoCA-T scores (0–22) were converted to standard MoCA scores (0–30).

§ Higher scores indicate better health.

PICLP was delivered in the four stages outlined in the panel, with good adherence to the service manual (appendix p 7). Details of the patients’ problems and the care provided by the PICLP clinicians have previously been published and are summarised briefly below.^18^ In the stage 1 assessment, the PICLP clinicians found that most patients had complex biopsychosocial problems (appendix pp 8–9). The majority of these problems were judged to be impediments to discharge (appendix p 9) and were prioritised in the stage 2 action plans. In stage 3, the interventions included working systemically with the ward team, patients, families, and medical and social out-of-hospital care providers to implement biopsychosocial care and discharge plans; advising about psychiatric aspects of care and prescribing; and delivering psychological interventions to patients and families (appendix p 10). In stage 4, the most common activity was advising the patients’ primary care physicians about post-discharge care. Few patients were referred to community psychiatric services at discharge (appendix p 10). The psychiatrists spent a mean of 71 min (SD 42) delivering PICLP to each patient (including the completion of workbooks and all other clinical records) and the assisting clinicians a mean of 75 min (SD 70).

In usual care, only 50 (3·6%) of 1371 participants were referred to the traditional consultation-liaison psychiatry services during their hospital stay.

**Figure 2:**
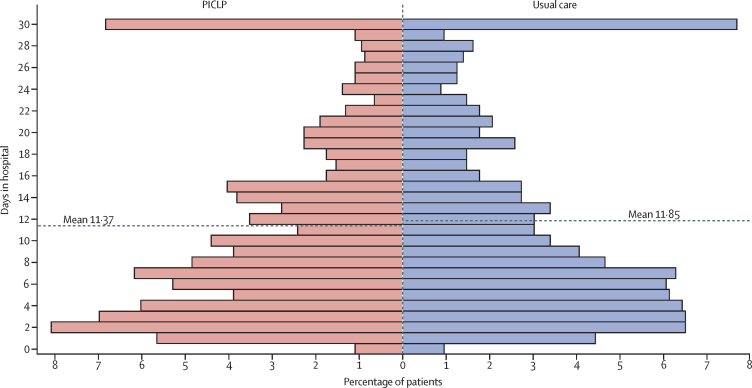
Primary outcome Comparison of the distributions of participants’ number of days in hospital in the 30 days after random allocation to PICLP or usual care. Data are unadjusted. PICLP=proactive integrated consultation-liaison psychiatry.

The mean time spent as an inpatient in the 30 days post-randomisation was 11·37 days (SD 8·74) with PICLP and 11·85 days (SD 9·00) with usual care (table 2). 91·7% of these days occurred during the index admission and 8·3% occurred during readmissions (appendix p 12). The estimated mean difference in days (–0·45 [95% CI –1·11 to 0·21]; p=0·18) in favour of PICLP was smaller than the 1-day difference sought, and not statistically significant (figure 2). The differences in treatment effect in the subgroup analyses of the primary outcome by hospital, sex, and age were not statistically significant (appendix p 13).

**Table 2:** The HOME Study outcomes

		**PICLP (n=1373)**	**Usual care (n=1371)**	Adjusted treatment effect estimate (95% CI)*	**p value**
**Primary outcome**					
Number of days spent as an inpatient in the 30 days post-randomisation†‡, mean (SD)		11·37 (8·74)	11·85 (9·00)	Difference between means −0·45 (−1·11 to 0·21)	0·18
**Secondary outcomes**					
Rate of discharge from hospital for the total length of the index admission§		NA	NA	RR 1·09 (1·00 to 1·17)	0·042
Discharge destination was a private residence¶, n (%)		843/1120 (75·3%)	802/1093 (73·4%)	OR 1·12 (0·91 to 1·38)	0·29
Length of the index admission (post-randomisation) truncated at 30 days§, mean (SD)		10·40 (8·63)	10·98 (8·83)	Difference between means −0·53 (−1·17 to 0·12)	0·11
Number of emergency readmissions to hospital in the year post-randomisation‡, mean (SD)		1·04 (1·77)	1·00 (1·61)	Mean count ratio 1·02 (0·91 to 1·14)	0·75
Number of days spent as an inpatient in the year post-randomisation‡, mean (SD)		21·31 (23·48)	21·41 (22·33)	Difference between means −0·17 (−1·87 to 1·53)	0·84
Rate of death in the year post-randomisation		NA	NA	RR 0·91 (0·81 to 1·03)	0·12
Number of emergency readmissions to hospital in the year post-randomisation§					
	Before the start of the COVID-19 pandemic	NA	NA	Mean count ratio 1·38 (0·76 to 2·52)	0·29
	After the start of the COVID-19 pandemic	NA	NA	Mean count ratio 0·82 (0·31 to 2·12)	0·68
Number of days spent as an inpatient in the year post-randomisation‖**					
	Before the start of the COVID-19 pandemic	NA	NA	Difference between means 0·40 (−1·55 to 2·34)	0·69
	After the start of the COVID-19 pandemic	NA	NA	Difference between means −0·21 (−4·88 to 4·46)	0·93
Rate of death in the year post-randomisation before the start of the COVID-19 pandemic**		NA	NA	RR 0·88 (0·76 to 1·00)	0·055
Experience of hospital stay (0–10), mean (SD)		8·15 (2·02)	8·08 (2·05)	Difference between means 0·05 (−0·13 to 0·22)	0·64
View on length of hospital stay					
	Too short	131 (9·5%)	120 (8·8%)	..	..
	About right	601 (43·8%)	592 (43·2%)	..	..
	Too long	302 (22·0%)	266 (19·4%)	..	..
	Comparison	..	..	Cumulative OR 1·05 (0·88 to 1·25)	0·57
Anxiety (GAD-2, 0–6), mean (SD)					
	1 month	2·03 (2·14)	1·94 (2·13)	Difference between means 0·11 (−0·06 to 0·28)	0·20
	3 months	1·79 (2·07)	1·69 (2·00)	Difference between means 0·11 (−0·07 to 0·29)	0·24
Depression (PHQ-2, 0–6), mean (SD)					
	1 month	2·03 (2·14)	1·90 (2·09)	Difference between means 0·10 (−0·07 to 0·27)	0·27
	3 months	1·84 (2·06)	1·63 (1·98)	Difference between means 0·20 (0·01 to 0·38)	0·035
Cognitive function (MoCA-T, 0–30), mean (SD)					
	1 month	16·99 (7·74)	16·94 (8·01)	Difference between means 0·21 (−0·30 to 0·73)	0·41
	3 months	17·97 (7·83)	18·23 (8·03)	Difference between means −0·20 (−0·79 to 0·38)	0·49
Independent functioning (Barthel, 0–100), mean (SD)					
	1 month	59·89 (30·64)	59·64 (30·64)	Difference between means −0·94 (−2·93 to 1·05)	0·36
	3 months	62·93 (30·66)	63·43 (30·43)	Difference between means −1·06 (−3·27 to 1·15)	0·35
Health-related quality of life (EQ–5D–5L), mean (SD)					
	1 month	0·35 (0·35)	0·35 (0·35)	Difference between means 0·00 (−0·03 to 0·02)	0·82
	3 months	0·34 (0·35)	0·34 (0·36)	Difference between means 0·00 (−0·02 to 0·03)	0·91
Overall quality of life (0–10), mean (SD)					
	1 month	5·92 (2·44)	5·93 (2·46)	Difference between means −0·05 (−0·25 to 0·16)	0·64
	3 months	6·26 (2·36)	6·40 (2·32)	Difference between means −0·15 (−0·36 to 0·07)	0·18

All comparisons are PICLP versus usual care. Barthel=Barthel Index of Activities of Daily Living. EQ-5D-5L=five-level EuroQol five-dimension questionnaire. GAD-2=Generalized Anxiety Disorder-2. MoCA-T=Montreal Cognitive Assessment-Telephone version. NA=not applicable. OR=odds ratio. PHQ-2=Patient Health Questionnaire-2. PICLP=proactive integrated consultation-liaison psychiatry. RR=rate ratio.

* Adjusted for hospital, sex, age, and medical ward; anxiety, depression, cognitive function, independent functioning, health-related quality of life, and overall quality of life were also adjusted for the corresponding baseline scores.

† During the index admission and any emergency readmissions to acute general hospitals.

‡ For PICLP, n=1359; for usual care, n=1351.

§ For PICLP, n=1373; for usual care, n=1370.

¶ For participants admitted from a private residence.

‖ During emergency admissions to acute general hospitals.

** Sensitivity analyses to address effects of the COVID-19 pandemic.

The rate of discharge for the total length of the index admission was 8·5% higher for participants allocated to PICLP than for those allocated to usual care, and this difference was statistically significant (rate ratio [RR] 1·09 [95% CI 1·00–1·17]; p=0·042). Inspection of the Kaplan–Meier plot (appendix p 14) indicates that the lines representing PICLP and usual care appear to separate at around 2 weeks post-randomisation. There were no statistically significant differences between PICLP and usual care in discharge destination or length of the index admission truncated at 30 days (table 2). Nor were there differences in number of emergency readmissions or number of days in hospital in the year after random allocation, in both our main analyses and those which sought to address effects of the COVID-19 pandemic.

1116 (40·7%) of the 2744 participants died in the year after random allocation (see appendix p 11 for causes of death). The Kaplan–Meier plot (appendix p 15) suggests a lower death rate for those allocated to PICLP, with the lines appearing to separate after 30 days post-randomisation, but the difference was not statistically significant (RR 0·91 [95% CI 0·81–1·03]; p=0·12). This effect of PICLP was slightly larger for deaths that occurred before the onset of the COVID-19 pandemic (RR 0·88 [0·76–1·00]; p=0·055).

At 3 months, the mean depression score was slightly higher with PICLP than with usual care (0·20 [95% CI 0·01–0·38]; p=0·035). There were no other statistically significant differences between PICLP and usual care on the outcomes collected from participants (table 2).

The mean cost of delivering PICLP was £207 (95% CI 200–214) per participant (appendix p 21). We found no meaningful differences in QALYs between PICLP and usual care (table 3). We estimated PICLP to be cost-saving compared with usual care over the 1 month time horizon (mean reduction £35 per patient; probability of cost-saving 60%) and the 3 month time horizon (mean reduction of £42 per patient; probability of cost-saving 55%; table 3, appendix p 21). We also estimated PICLP to be cost-effective over the 1 month and 3 month time horizons for thresholds of £20 000 per QALY or less, but it was dominated by usual care (more costly and less effective) over a 12 month time horizon.

**Table 3:** The HOME Study health economic outcomes

	**PICLP (n=1373)**	**Usual care (n=1371)**	**Difference (95% CI)**	**ICER**	**Probability that PICLP is cost-saving**	**Probability that PICLP is cost-effective**		
						At £15 000 per QALY threshold	At £20 000 per QALY threshold	At £30 000 per QALY threshold
**To 1 month (30 days) time horizon**								
Total cost of inpatient care*	£5152	£5187	−£35 (−392 to 322)	£77 717 (SW)†	60%	59%	58%	57%
QALYs	0·0264	0·0268	−0·0004 (−0·0015 to 0·0006)	..	..	..	..	..
**To 3 months (90 days) time horizon**								
Total cost of inpatient care*	£8100	£8143	−£42 (−724 to 640)	£22 191 (SW)†	55%	52%	51%	48%
QALYs	0·0857	0·0876	−0·0019 (−0·0067 to 0·0029)	..	..	..	..	..
**To 12 months (365 days) time horizon**								
Total cost of inpatient care*	£14 041	£13 921	£120 (−1036 to 1277)	Dominated‡	43%	38%	36%	35%
QALYs§	0·3260	0·3312	−0·0052 (−0·0280 to 0·0176)	..	..	..	..	..

All comparisons are PICLP versus usual care. ICER=incremental cost-effectiveness ratio (incremental cost per QALY). PICLP=proactive integrated consultation-liaison psychiatry. QALY=quality-adjusted life year. SW=southwest quadrant.

* Comprises the cost of PICLP delivery (mean £207 [95% CI 200 to 214]) based on the time consultants and assisting clinicians took to deliver the intervention, the index admission, and subsequent emergency admissions to acute general hospitals.

† An intervention with an ICER in the southwest quadrant of the cost-effectiveness plane (cost-saving and less effective) is cost-effective if the ICER is above the cost-effectiveness threshold.

‡ Dominated means more costly and less effective than usual care.

§ Derived by extrapolation (assumes constant utility after 3 months for those alive beyond this timepoint).

There were 323 serious adverse events (158 deaths in the 30 days post-randomisation in the PICLP group *vs* 165 in the usual care group), none of which were judged to be related to the trial treatments or procedures.

## Discussion

In this large, randomised controlled trial, we studied the effectiveness and cost-effectiveness of PICLP-enhanced care in reducing the time that older patients spend in acute medical wards. The 2744 participants had severe and complex biopsychosocial problems with a high prevalence of cognitive impairment, symptoms of depression and anxiety, functional dependency, and medical multimorbidity. This complexity is typical of the older medical inpatient population in England. We found that participants allocated to PICLP spent a mean of 0·45 fewer days in hospital in the 30 days after random allocation than those allocated to usual care. This difference was smaller than the 1-day difference sought and not statistically significant, with confidence intervals extending from a reduction of 1·1 days to an increase of 0·2 days. The rate of discharge for the total length of the index admission was higher with PICLP—a difference most apparent in those patients who stayed in hospital for more than 2 weeks. We also observed some evidence of a lower death rate over the year after randomisation with PICLP, a difference that appeared to be larger for deaths before the onset of the COVID-19 pandemic. On outcomes collected from participants, the only statistically significant difference was a slightly higher mean depression score with PICLP at 3 months, which is of doubtful clinical significance. In our health economic analysis, we found that PICLP was likely to be modestly cost-saving compared with usual care over the 1 month and 3 month (but not 12 month) time horizons. Similarly, we estimated it to be cost-effective over these time horizons at thresholds of £20 000 per QALY or lower.

Why did we not find a larger and statistically significant effect of PICLP on our primary outcome? We propose a number of possible reasons. First, our conceptualisation of how PICLP could reduce time in hospital might have been inadequate. We expected that it would help ward teams to manage biopsychosocial complexity and thereby reduce time in hospital. In qualitative interviews, patients and ward staff reported that it did help with the management of complexity.^19^ However, the PICLP clinicians described additional obstacles to prompt discharge that they found difficult to overcome.^18^ One was difficulty in achieving a ward team consensus that a patient could go home; in the PICLP clinicians’ view, other ward staff often overestimated the risk of discharge and underestimated the risk of staying in hospital. Another was difficulty in arranging adequate and timely out-of-hospital social care for those patients who needed help with daily tasks. Second, the intensity of PICLP might have been suboptimal. Although we observed good adherence to the service manual, the PICLP clinicians spent only a modest amount of time delivering it. This made PICLP relatively inexpensive, but it could have impaired its potential effectiveness. Third, the trial included patients unlikely to benefit from PICLP. Many patients had relatively short hospital stays, whereas our findings suggest that PICLP might be more effective in achieving discharge for those with longer stays. Fourth, trial procedures could have inadvertently impaired the effectiveness of PICLP. The PICLP clinicians aimed to proactively assess patients as soon as possible after their admission to the medical ward. However, this assessment was delayed for an average of 2 days by the trial enrolment procedures, restricting the influence of PICLP on patients’ initial management plans. Additionally, PICLP clinicians reported that working in the context of an individually randomised trial sometimes made it difficult for them to be fully integrated into the ward team.^18^ Fifth, there could have been contamination of usual care. There was no evidence of increased referrals to traditional consultation-liaison psychiatry in usual care, but the daily presence of the PICLP clinicians on the wards could have changed practice by increasing ward team members’ awareness of psychosocial problems and the negative consequences of prolonged hospital stays. Sixth, the trial was underpowered to detect the 1-day difference in time in hospital sought because recruitment was curtailed by the COVID-19 pandemic. Although our best estimate of the treatment effect is a 0·45-day difference, the confidence intervals around this estimate are wide and include the 1-day difference we sought—thus, this trial cannot exclude a treatment effect of this size.^29^, ^30^

The lower death rate observed with PICLP over the year after random allocation was an unexpected finding. Although it should be interpreted with caution, because it was only of borderline statistical significance for deaths before the onset of the COVID-19 pandemic, this difference is clinically plausible. The PICLP clinicians reported that they helped patients and families to understand their medical, as well as psychiatric, diagnoses and treatments.^18^ This might have had lasting positive effects on their engagement with medical care.

Our finding that PICLP is likely to be cost-saving in the short-term indicates that the benefits reported by patients and ward staff could come at no additional cost to the hospital (assuming that the hospital pays for both medical and psychiatric care).^19^ The estimated cost-effectiveness of PICLP over 1 month and 3 month time horizons, but not a 12 month time horizon, is consistent with PICLP being provided for only 30 days after random allocation; its implementation for a longer period might have yielded different findings. Given that the difference in the primary outcome was not statistically significant, it might seem surprising that PICLP is likely to be cost-effective. However, such apparently contradictory conclusions are not uncommon, and reflect the differing perspectives and methods of statistical significance testing and health economic decision analysis.^31^

The study limitations, in addition to those described above, include the following: uncertain generalisability to other patient populations, hospitals, and health-care systems; restriction of the primary outcome to the time spent in hospital in the 30 days post-randomisation; the use of proxies to provide some of the secondary outcome data; extrapolation of health-related quality of life scores beyond 3 months; no data on the effects of PICLP on time spent by other ward staff on patient care; and an economic analysis that took an acute hospital perspective and did not include costs to social and community care services, or to patients and their families.

The HOME Study is, to the best of our knowledge, the first randomised controlled trial of proactive and integrated consultation-liaison psychiatry, and the first study of its effectiveness in older adults.^22^ Although a recent systematic review reported that proactive and integrated consultation-liaison psychiatry services reduce time in hospital for working-age adults, this evidence was restricted to non-randomised studies.^15^

The HOME Study has implications for the design and conduct of future trials in the older medical inpatient population. It shows that large clinical trials with representative samples can be done, despite the severity and complexity of these patients’ problems. We found that patients were willing to participate, and the use of consultees allowed even those with severe cognitive impairment to do so. Furthermore, we found that high follow-up rates can be achieved by tailoring procedures to the needs of this population. It also highlights the need to carefully consider the choice of primary outcome. We chose time spent in hospital. However, the wide variability of this measure necessitated a very large sample size. A trial adequately powered to detect the half-day difference we found, which may still be clinically meaningful, would require a sample of more than 10 000 patients.

The findings of The HOME Study, as reported here and in associated publications, indicate that PICLP can be delivered at scale, is experienced by older medical inpatients and ward staff as enhancing medical care, and could be cost-saving in the short-term.^19^ However, the results provide insufficient evidence to recommend PICLP's implementation for the purpose of reducing time in hospital. We conclude that the further development of PICLP to achieve more intensive delivery, a sharper focus on patients at very high risk of longer stays, and greater influence on both inpatient and out-of-hospital care, is both warranted and needed.

### Contributors

### Full collaborating author list

### Data sharing

The protocol, statistical analysis plan, Proactive Integrated Consultation-Liaison Psychiatry (PICLP) manual (appendix pp 26–39), and PICLP workbook are all freely available (appendix pp 40–48). Individual participant data cannot be fully anonymised and therefore will not be shared.

## Declaration of interests

We declare no competing interests.
