# Supplementary material for: Proactive integrated consultation-liaison psychiatry and time spent in hospital by older medical inpatients in England (The HOME Study): a multicentre, parallel-group, randomised controlled trial
Source: Lancet Psychiatry. 2024 Sep;11(9):684–95. doi: 10.1016/S2215-0366(24)00188-3 (PMC11469986; doi:10.1016/S2215-0366(24)00188-3)
Supplement: Supplementary appendix [file mmc1.pdf]

THE LANCET
Psychiatry

Supplementary appendix

This appendix formed part of the original submission and has been peer reviewed. We post it as supplied by the authors.

Supplement to: Sharpe M, Walker J, van Niekerk M, et al. Proactive integrated consultation-liaison psychiatry and time spent in hospital by older medical inpatients in England (The HOME Study): a multicentre, parallel-group, randomised controlled trial. *Lancet Psychiatry* 2024; published online Aug 10. https://doi.org/10.1016/S2215-0366(24)00188-3.

Supplementary Appendix

Contents

Study Team, Collaborators and Trial Oversight Committees 3
Full List of Authors 4
Additional baseline data 5
Additional data about the index admission 6
Adherence to the PICLP service manual 7
Biopsychosocial problems identified at the PICLP Stage 1 assessment 8
Number of problem categories per patient at the PICLP Stage 1 biopsychosocial assessment 9
Interventions delivered by the PICLP clinicians 10
Causes of death in the year post-randomisation 11
Percentage of participants in hospital during their index admission, in hospital during an emergency readmission, alive and not in hospital, and dead on each of the 30 days post-randomisation. 12
Subgroup analyses of the primary outcome 13
Rate of discharge for the total length of the index admission 14
Rate of death in the year post-randomisation 15
Data collection from participants 16
Bootstrap 95% confidence intervals for outcomes analysed using linear regression 16
Patient-centred analyses of the primary outcome 17
Patient-centred analyses of readmissions and time in hospital in the year post-randomisation 17
Effects of proxy data collection on relevant secondary outcomes 18
Details of the sensitivity analyses to address effects of the COVID-19 pandemic 19
Unit costs 20
Additional cost-effectiveness data 21
Data coding and databases 22
Supplementary post-hoc analyses 24
PICLP service manual 26
PICLP clinicians' workbook 40

Study Team, Collaborators and Trial Oversight Committees

Trial Management: Michael Sharpe (Chief Investigator), Jane Walker (Trial Management Lead), Mark Toynbee, Maike van Niekerk, Katy Burke, Chris Frost, Nicholas Magill, Tobit Emmens, Chris Dickens, Gloria Calderon, Annabel Price, Vicki Barber.

Principal Investigators and number of participants recruited from each site: Colm Owens and Chris Dickens (Exeter; 1,273), Jane Walker (Oxford; 1,058), Annabel Price (Cambridge; 413).

Recruitment and baseline data collection:
Maike van Niekerk, Rhian Bold, Lucy McIvor, Chloe Stokes, Elliot Hampsey, Will Turner, Sophie Howitt, Hannah Chaitow, Georgiana Jennings, Jennifer Mbanu, Kate Huggett, Nicola Walker, Stacey Horne, Iona Cairns, Tanja Christensen, Sian Lison, Davin Turner, Grace Parker, Daniel Scott, Julie Philps, Rhian Kant, Katie Keating-Fedders, Shelley Campbell.

PICLP delivery: Michael Sharpe, Luke Solomons, Michael Yousif, Tom Bajorek, Mark Toynbee, Gabrielle Sirois-Giguere, Jessica Harris, Colm Owens, Aelfrida Palmer, Amanda Henderson, Jonathan Burns, Laura Hill, Annabel Price, Felix Clay, Myles Doyle, Gunes Berk.

Outcome data collection and data entry: Michael Daly, Ben Steward, Katy Burke, Laura Hollands, Harriet Hobbs, Ellie Macey, Naomi Hannaway, George Kitchen, Timur Ohloff, Louise Pollard, Ruth Harman, Anna Scholcz, Isabelle Rocroi.

Statistics: Chris Frost, Nicholas Magill, Ian White

Health Economics: Simon Walker, Ana Duarte.

Collaborators: Paul Brennan, Bart Sheehan, Ray Fitzpatrick, Sasha Shepperd, Tjeerd Van Staa, Alistair Burns, Sallie Lamb.

Trial Steering Committee: Amanda Ramirez (Chair), Peter White, Hywel Jones, Chris Griffiths, Paul McCrone, Aileen Clarke, Mark Mullee, Thomas Jackson.

Data Monitoring Committee: Matthew Hotopf (Chair), Sabine Landau, Tomas Welsh.

Patient and Public Involvement Panel: Gillian Skyte, Francis Josephs, Karen Swaffield, Sylvia Bailey, Deirdre Jalie, Julia Hamer Hunt, Michael Worthington.

Full List of Authors

Psychological Medicine Research, University of Oxford Department of Psychiatry, Warneford Hospital, Oxford, UK (M Sharpe MD, J Walker PhD, M van Niekerk DPhil, M Toynbee DPhil, R Bold MBBS, H Hobbs MSc, S Howitt MSc, E Macey BSc, L Pollard MSc, I Rocroi BA); **Department of Medical Statistics, London School of Hygiene and Tropical Medicine, London, UK** (N Magill PhD, C Frost MA); **MRC Clinical Trials Unit at UCL, London, UK** (IR White PhD); **Centre for Health Economics, University of York, York, UK** (S Walker MSc, A Duarte MSc); **NHS Devon Mental Health, Learning Disability and Neurodiversity Provider Collaborative, Devon, UK** (C Owens MBChB); **University of Exeter, Exeter, UK** (C Dickens PhD, J Burns PhD, L Hollands BSc, S E Lamb DPhil)**; Cambridgeshire and Peterborough NHS Foundation Trust, Cambridge, UK** (A Price PhD, G Berk MD, S Campbell MSc, F Clay MBBS, J Philps BSc)**; Sussex Partnership Foundation Trust, Worthing, UK** (P Aitken MBChB)**; Oxford University Hospitals NHS Foundation Trust, Oxford, UK** (T Bajorek BMBCh, H Chaitow BSc, A Scholcz BA, L Solomons MBBS, W Turner MSci)**; Central and North West London Foundation Trust and University College London Hospitals Foundation Trusts, London, UK** (K Burke MBBS)**; University of Bristol, Bristol, UK (**M Daly PhD**); Devon Partnership NHS Trust, Devon, UK** (T Emmens MSc, L Hill MBBS, A Palmer MSc)**; King's College, London, London, UK** (E Hampsey MSc)**; University College London, London, UK** (N Hannaway MSc)**; Gloucestershire Health and Care NHS Foundation Trust, Gloucester, UK** (J Harris MBChB)**; University of Nottingham, Nottingham, UK** (R Harwood MD)**; University of Bath, Bath, UK (**R Kant MSc**); University of Warwick, Coventry, UK** (D Lasserson MD)**; University of Rochester School of Medicine, Rochester NY, USA** (H B Lee MD)**; Nuffield Department of Population Health, University of Oxford, Oxford, UK** (S Shepperd DPhil)**; Université de Sherbrooke, Quebec, QC, Canada** (G Sirois-Giguere MD)**; School of Medicine and Psychology, Australian National University, Canberra, ACT, Australia** (B Steward MSc)**; West London NHS Trust, London, UK** (M Yousif MSc)

Additional baseline data

	PICLP (n=1373)	Usual care (n=1371)
**Main reason for hospital admission**		
Cardio-respiratory symptoms	380 (28%)	374 (27%)
Falls & injuries	192 (14%)	204 (15%)
Gastrointestinal symptoms	166 (12%)	180 (13%)
Confusion, drowsiness & collapse	174 (13%)	159 (12%)
General weakness	88 (6%)	105 (8%)
Urinary symptoms	89 (6%)	81 (6%)
Fever and suspected infection	74 (5%)	69 (5%)
Cellulitis and ulcers	51 (4%)	61 (4%)
Neurological symptoms	43 (3%)	41 (3%)
Back and limb pain	27 (2%)	31 (2%)
Abnormal investigation findings	28 (2%)	14 (1%)
Other	61 (4%)	52 (4%)
**Medications prescribed**		
Antidepressant	348 (25%)	319 (23%)
Anxiolytic / hypnotic	242 (18%)	219 (16%)
Dementia medication	74 (5%)	89 (6%)
Antipsychotic	89 (6%)	70 (5%)
Lithium	8 (1%)	4 (<1%)
**Anticholinergic burden score** *Median, range*	1, 0-14	1, 0-11
**Socioeconomic deprivation**		
1st quintile (most deprived)	39 (3%)	36 (3%)
2nd quintile	161 (12%)	164 (12%)
3rd quintile	309 (23%)	319 (23%)
4th quintile	404 (29%)	360 (26%)
5th quintile (least deprived)	460 (34%)	492 (36%)
**Residence area**		
Urban	794 (58%)	775 (57%)
Rural	579 (42%)	596 (43%)
**Consent procedure**		
Participant gave informed consent	810 (59%)	813 (59%)
Personal consultee agreed	530 (39%)	526 (38%)
Nominated consultee agreed	33 (2%)	32 (2%)

If a patient lacked capacity, we identified a consultee who could advise on whether they should be enrolled in the trial (in accordance with the Mental Capacity Act 2005, England and Wales). Where possible this was a personal consultee (family member, carer or friend). If a personal consultee could not be identified a nominated consultee was asked to advise (a senior clinician with no connection to the research).

Additional data about the index admission

	PICLP (n=1373)	Usual care (n=1371)
**Recorded incidents**		
Number of incidents *Median, range*	0, 0-3	0, 0-3
Participants with ≥1 recorded incident	67 (5%)	60 (4%)
**Medications prescribed at discharge**		
Number prescribed *Median, range*	8, 1-26	9, 1-29
Antidepressant	351 (26%)	305 (22%)
Anxiolytic / hypnotic	202 (15%)	181 (13%)
Dementia medication	98 (7%)	86 (6%)
Antipsychotic	95 (7%)	86 (6%)
Lithium	7 (1%)	4 (<1%)
**Anticholinergic burden score at discharge *Median, range***	1, 0-11	1, 0-10
**Length of hospital admission (days) *Mean (SD)***		
From randomisation	11·2 (11·2)	12·1 (12·6)
From admission	14·7 (11·4)	15.5 (12·8)
**Reason for end of hospital admission**		
Discharged to private residence	876 (64%)	844 (62%)
Discharged to care / nursing home	266 (19%)	246 (18%)
Discharged to other location	126 (9%)	150 (11%)
Died	105 (8%)	130 (9%)

Adherence to the PICLP service manual

Measure	Adherence
Biopsychosocial assessment completed	1357/1373 (99%)
Biopsychosocial assessment completed within 1 day of allocation	1312/1359 (97%)
All problem categories assessed	1359/1359 (100%)
Action plan made	1359/1359 (100%)
Action plan discussed with medical ward team	1307/1359 (96%)
Checklist fully completed	1359/1359 (100%)
Progress reviewed every weekday	924/1359 (68%)*

PICLP adherence was measured using data from the PICLP checklists. *Progress reviewed every working weekday; if only one working day missed 1307/1359 (96%).

Biopsychosocial problems identified at the PICLP Stage 1 assessment

Domain	Problem category	Examples of problems / diagnoses in this category	Patients with at least 1 problem in this category n (%)	Patients with at least 1 problem in this category impeding discharge n (%)
**Biomedical**	Not medically safe for discharge	Active medical conditions requiring urgent treatment	1103 (81)	1089 (80)
	Sensory deficits	Impaired hearing or eyesight	436 (32)	58 (4)
	Medication-related problems	Non-concordance, polypharmacy, side-effects	316 (23)	235 (17)
**Psychological**	Cognitive impairment	Delirium with or without dementia	737 (54)	487 (36)
	Other psychiatric conditions	Depression, anxiety*	291 (21)	165 (12)
	Psychological symptoms	Fear of falling, distress	318 (23)	167 (12)
	Behavioural problems	Agitation, aggression	185 (14)	140 (10)
	Substance misuse	Misuse of alcohol	118 (9)	64 (5)
**Social**	Dependency in basic activities of daily living	Unable to walk without assistance	1141 (84)	969 (71)
	Dependency in instrumental activities of daily living	Unable to drive or go shopping without assistance	1004 (74)	891 (66)
	Legal problems	Unclear capacity to make treatment or discharge decisions	256 (19)	108 (8)
	Accommodation problems	Accommodation isolated or inappropriate to needs	250 (18)	192 (14)

Four patients had no problems and 13 had problems but none impeding discharge (all 17 were discharged soon after assessment). * Other psychiatric diagnoses e.g. schizophrenia, bipolar disorder were uncommon.

Number of problem categories per patient at the PICLP Stage 1 biopsychosocial assessment

n = 1359	Number of categories in which each patient had a problem (0-12)	Number of categories in which each patient had a problem impeding discharge (0-12)
Mean (SD)	4·5 (1·8)	3·4 (1·7)
Median (range)	5 (0-11)	3 (0-9)
0	4 (<1%)	17 (1%)
1	66 (5%)	182 (13%)
2	119 (9%)	242 (18%)
3	202 (15%)	304 (22%)
4	288 (21%)	296 (22%)
5	286 (21%)	176 (13%)
6	200 (15%)	83 (6%)
7	120 (9%)	48 (4%)
8	49 (4%)	10 (<1%)
9	20 (2%)	1 (<1%)
10	4 (<1%)	0 (0%)
11	1 (<1%)	0 (0%)
12	0 (0%)	0 (0%)

Interventions delivered by the PICLP clinicians

Intervention category	n (%)
**Stage 3:**	
Regular communication with ward team	1245 (92)
Focussed discharge planning with ward team	1233 (91)
Focussed discharge planning with patient	1061 (78)
Driving implementation of management plan	1050 (77)
Advice to ward team about psychiatric diagnoses	825 (61)
Focussed discharge planning with family and friends	742 (55)
Routine board round discussions	660 (49)
Advice to ward team about environmental and functional optimisation	633 (47)
Advice to ward team about medications	627 (46)
Psychological interventions with patient	623 (46)
Advice to ward team about psychological and behavioural interventions	554 (41)
Advice to ward team about investigations	477 (35)
Participating in multidisciplinary team meetings	443 (33)
Focussed discharge planning with hospital staff other than ward team	397 (29)
Participating in discussions with other medical specialties	343 (25)
Psychological interventions with the patient's family	314 (23)
Focussed discharge planning with out-of-hospital services	129 (10)
Seeing patient jointly with ward team members	124 (9)
Advice to ward team about risk minimisation on ward	119 (9)
Focussed discharge planning with paid carers	113 (8)
Psychological interventions to ward team	89 (7)
Advice to ward team about the use of mental health legislation	86 (6)
**Stage 4:**	
Advice to primary care physician	384 (28)
Referral to community psychiatry	97 (7)
Advice to other community healthcare professionals e.g. palliative care	90 (7)
Advice to other out-of-hospital professionals e.g. social services	42 (3)

N = 1359 patients

Causes of death in the year post-randomisation

	PICLP (n=542)	Usual care (n=574)
**Cancer**		
Gastro-intestinal	22 (4%)	26 (5%)
Lung (including mesothelioma)	18 (3%)	29 (5%)
Prostate	17 (3%)	21 (4%)
Hepato-biliary	13 (2%)	16 (3%)
Unknown primary	10 (2%)	15 (3%)
Other cancer	48 (9%)	34 (6%)
**Circulatory**		
Ischaemic heart disease (including acute myocardial infarction)	49 (9%)	51 (9%)
Cerebrovascular disease (stroke)	27 (5%)	23 (4%)
Heart valve disease	11 (2%)	19 (3%)
Arrythmia	8 (1%)	7 (1%)
Heart failure	3 (1%)	5 (1%)
Pulmonary embolism	2 (0%)	0 (0%)
Cardiomyopathy	0 (0%)	4 (1%)
Other circulatory	14 (3%)	18 (3%)
**Respiratory**		
Respiratory infection	40 (7%)	47 (8%)
Chronic obstructive airways disease	35 (6%)	33 (6%)
Interstitial pulmonary disease	7 (1%)	12 (2%)
Other respiratory	14 (3%)	10 (2%)
**Neurological & psychiatric**		
Dementia	70 (13%)	60 (10%)
Parkinson disease	14 (3%)	14 (2%)
Degenerative neurological disease (including motor neuron disease)	8 (1%)	5 (1%)
Multiple sclerosis	1 (0%)	5 (1%)
**Other**		
Gastro-intestinal and hepato-biliary diseases	30 (6%)	34 (6%)
COVID-19	12 (2%)	14 (2%)
Other (non-respiratory) infection	19 (4%)	18 (3%)
Senility	12 (2%)	10 (2%)
Diabetes	8 (1%)	5 (1%)
Fall or injury	9 (2%)	11 (2%)
Renal disease	2 (0%)	7 (1%)
Other	15 (3%)	19 (3%)
**Unknown**	4 (1%)	2 (0%)

Percentage of participants in hospital during their index admission, in hospital during an emergency readmission, alive and not in hospital, and dead on each of the 30 days post-randomisation.

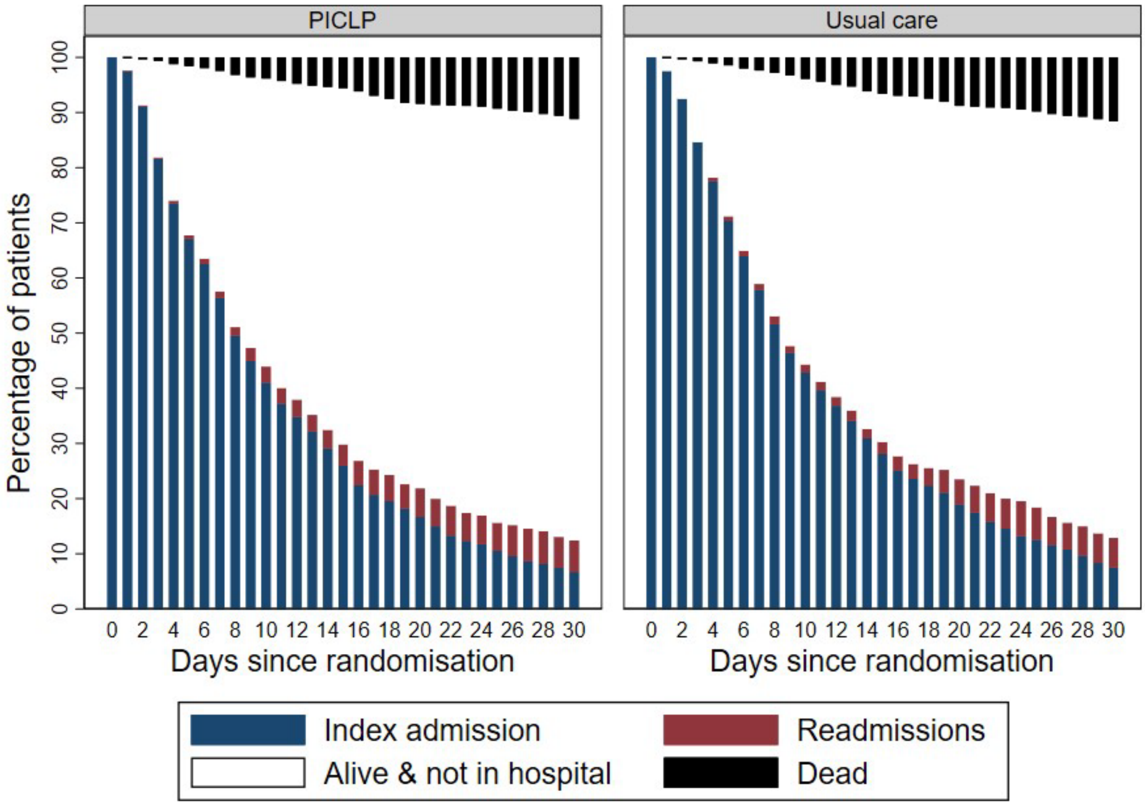

Subgroup analyses of the primary outcome

Moderator		PICLP (n=1373)	Usual care (n=1371)	Adjusted difference between means	Test of differences in treatment effect
**Hospital**	Exeter	11·21 (8·55)	11·23 (8·60)	0·04 days<br>95% CI -0·93 to 1·01	p=0·30
	Oxford	11·34 (8·89)	12·24 (9·27)	-0·82 days<br>95% CI -1·90 to 0·25	
	Cambridge	11·91 (8·95)	12·74 (9·38)	-1·00 days<br>95% CI -2·71 to 0·72	
**Sex**	Male	11·59 (8·62)	12·16 (9·07)	-0·64 days<br>95% CI -1·57 to 0·29	p=0·57
	Female	11·14 (8·86)	11·53 (8·91)	-0·25 days<br>95% CI -1·20 to 0·69	
**Age**	65-74 years old	10·26 (8·49)	10·97 (8·81)	At age 70<br>-0·61 days<br>95% CI -1·81 to 0·60	p=0·76
	75-84 years old	11·86 (8·98)	12·29 (9·34)	At age 80<br>-0·48 days<br>95% CI -1·17 to 0·21	
	≥85 years old	11·56 (8·63)	11·95 (8·78)	At age 90<br>-0·35 days<br>95% CI -1·27 to 0·56	

Rate of discharge for the total length of the index admission

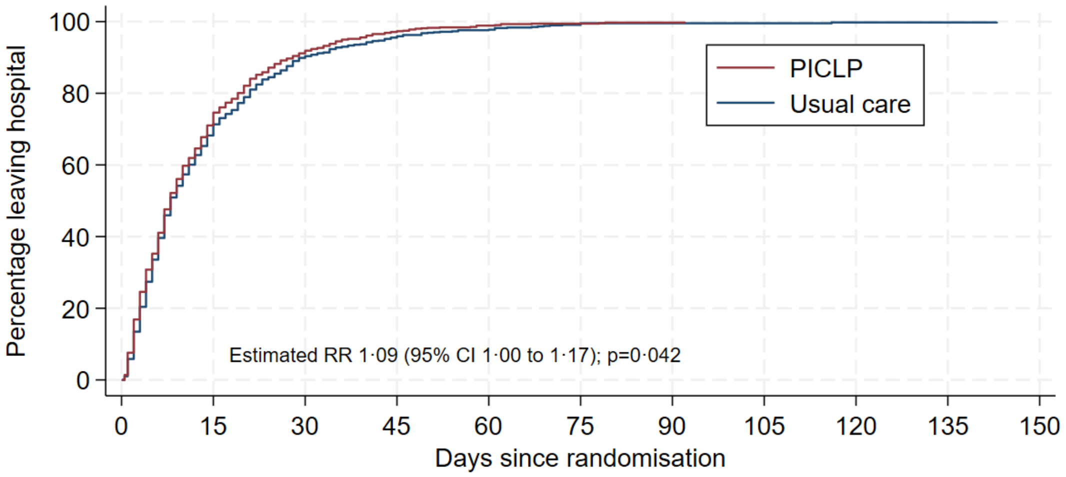

	0	15	30	45	60	75	90	105	120	135	150
**Usual care**											
At-risk	1371	388	104	38	17	7	3	3	2	2	1
Censored	0	75	110	124	129	129	130	130	130	130	130
Event	0	908	1157	1209	1225	1235	1238	1238	1239	1239	1240
**PICLP**											
At-risk	1373	363	95	25	8	4	1	0	0	0	0
Censored	0	63	92	102	104	104	105	105	105	105	105
Event	0	947	1186	1246	1261	1265	1267	1268	1268	1268	1268

Discharges per day amongst those alive and remaining in hospital

Rate of death in the year post-randomisation

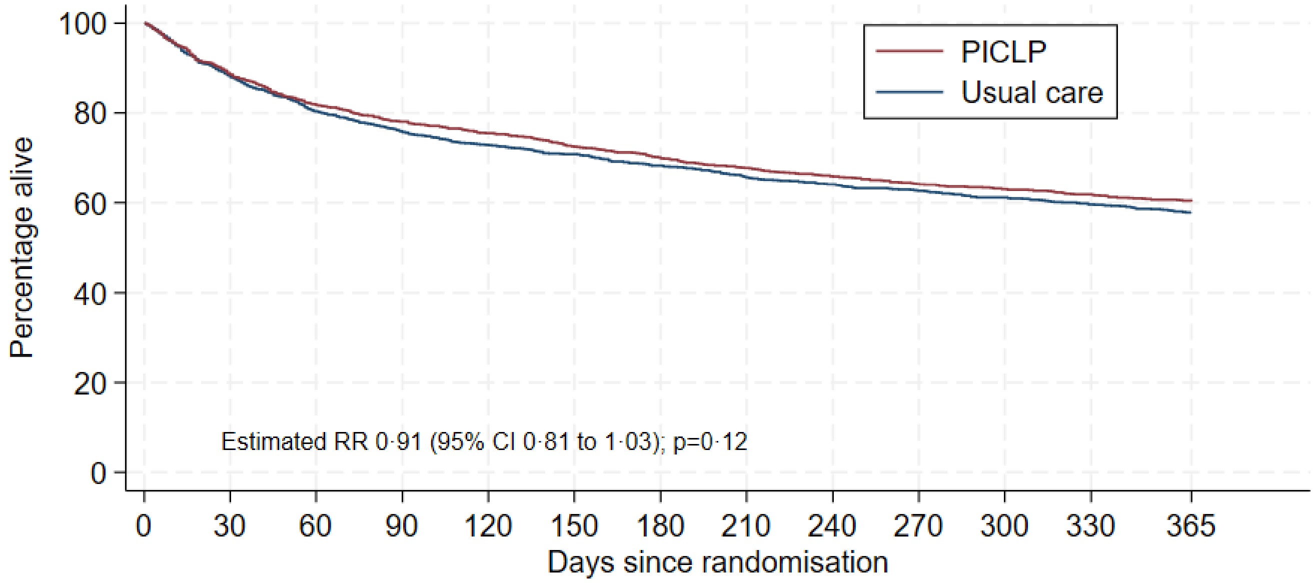

**Usual care**													
At-risk	1371	1209	1099	1038	987	957	923	889	866	847	826	805	781
Censored	0	3	4	4	14	16	16	16	16	16	16	16	16
Event	0	159	268	329	370	398	432	466	489	508	529	550	574
**PICLP**													
At-risk	1373	1219	1125	1072	1030	987	954	922	897	873	857	841	822
Censored	0	0	0	0	7	10	10	10	10	10	10	10	10
Event	0	154	248	301	336	376	409	441	466	490	506	522	541

Data collection from participants

	PICLP (n=1373)	Usual care (n=1371)
**1 month**		
Data obtained	1076 (78%)	1029 (75%)
Dead	199 (14%)	213 (16%)
Refused	69 (5%)	101 (7%)
Too unwell and no proxy	18 (1%)	15 (1%)
Uncontactable	11 (1%)	13 (1%)
**3 months**		
Data obtained	929 (68%)	868 (63%)
Dead	298 (22%)	331 (24%)
Refused	98 (7%)	132 (10%)
Too unwell and no proxy	27 (2%)	18 (1%)
Uncontactable	21 (2%)	22 (2%)

Bootstrap 95% confidence intervals for outcomes analysed using linear regression

Outcome	Estimate	Parametric 95% CI	Bootstrap 95% CI
Number of days spent as an inpatient in the 30 days post-randomisation (primary outcome)	-0·45 days	-1·11 to 0·21 days	-1·13 to 0·20 days
Length of the index admission (post-randomisation) truncated at 30 days	-0·53 days	-1·17 to 0·12 days	-1·17 to 0·14 days
Number of days spent as an inpatient in the year post-randomisation	-0·17 days	-1·87 to 1·53 days	-1·74 to 1·69 days
Experience of the hospital stay	0·05	-0·13 to 0·22	-0·14 to 0·21
Anxiety (GAD-2, 0-6)			
1 month	0·11	-0·06 to 0·28	-0·05 to 0·30
3 months	0·11	-0·07 to 0·29	-0·07 to 0·29
Depression (PHQ-2, 0-6)			
1 month	0·10	-0·07 to 0·27	-0·08 to 0·27
3 months	0·20	0·01 to 0·38	0·01 to 0·38
Cognitive function (MoCA-T, 0-30)			
1 month	0·21	-0·30 to 0·73	-0·32 to 0·70
3 months	-0·20	-0·79 to 0·38	-0·80 to 0·38
Independent functioning (Barthel, 0-100)			
1 month	-0·94	-2·93 to 1·05	-2·95 to 1·10
3 months	-1·06	-3·27 to 1·15	-3·27 to 1·33
Health-related quality of life (EQ-5D-5L)			
1 month	0·00	-0·03 to 0·02	-0·02 to 0·02
3 months	0·00	-0·02 to 0·03	-0·02 to 0·03
Overall quality of life (0-10)			
1 month	-0·05	-0·25 to 0·16	-0·23 to 0·16
3 months	-0·15	-0·36 to 0·07	-0·33 to 0·09

GAD-2 = Generalized Anxiety Disorder-2, PHQ-2 = Patient Health Questionnaire-2, MoCA-T = Montreal Cognitive Assessment-Telephone Version, Barthel = Barthel Index of Activities of Daily Living, EQ-5D-5L = EuroQol 5D

Patient-centred analyses of the primary outcome

In the main analysis of the primary outcome, a patient may be in hospital for fewer than 30 days because they have been discharged or because they have died. To complement this hospital-centred analysis, we conducted two supplementary analyses which took a more patient-centred approach. These considered time spent in hospital as a proportion of the time alive in the 30 days post-randomisation and time spent in hospital in the 30 days post-randomisation only including participants who were alive for all 30 days.

	**PICLP (n=1373)**	**Usual care (n=1371)**	**Adjusted difference between means**
Time spent in hospital as a proportion of the time alive in the 30 days post-randomisation	0·43 (0·33)	0·45 (0·34)	-0·02<br>95% CI -0·04 to 0·01, p=0·16<br>Bootstrap 95% CI -0·04 to 0·01

	**PICLP (n=1205**	**Usual care (n=1193)**	**Adjusted difference between means**
Time spent in hospital in the 30 days post-randomisation only including participants who were alive for all 30 days	11·42 (8·93)	11·94 (9·19)	-0·49 days<br>95% CI -1·21 to 0·23, p=0·18<br>Bootstrap 95% CI -1·19 to 0·25

Patient-centred analyses of readmissions and time in hospital in the year post-randomisation

To complement the hospital-centred analyses of readmissions and days in hospital in the year post-randomisation, we conducted two supplementary analyses which took a more patient-centred approach. These considered number of emergency readmissions to hospital in the year post-randomisation modelled using the number of readmissions scaled by time alive (measured in years), and time (days) spent in hospital in the year post-randomisation modelled using the number of days participants spent in hospital as a proportion of time alive.

	**PICLP (n=1373)**	**Usual care (n=1371)**	Mean count ratio PICLP:UC
Number of readmissions in the year post-randomisation, scaled by time alive	1·04 (1·77)	1·00 (1·61)	1·00<br>95% CI 0·89 to 1·12, p=0·94

	**PICLP (n=1373)**	**Usual care (n=1371)**	Adjusted difference between means
Days in hospital in the year post-randomisation as a proportion of time alive	0·21 (0·30)	0·22 (0·31)	-0·01<br>95% CI -0·03 to 0·01, p=0·32

Effects of proxy data collection on relevant secondary outcomes

	PICLP	Usual care	p-value for whether proxy measurements differ by group	p value for whether treatment effect differs by patient/ proxy
**Experience of the hospital stay**				
Patient	8·4 (1·9)	8·4 (1·9)	0·66	0·69
Proxy	7·6 (2·1)	7·4 (2·2)		
**View on the length of the hospital stay**				
Patient				
Too short	100 (14%)	82 (12%)	0·51	0·08
About right	472 (65%)	463 (67%)		
Too long	153 (21%)	141 (21%)		
Proxy				
Too short	31 (10%)	38 (13%)		
About right	129 (42%)	129 (44%)		
Too long	149 (48%)	125 (43%)		
**Anxiety at 1 month**				
Patient	1·7 (2·0)	1·6 (2·0)	0·97	0·36
Proxy	2·8 (2·3)	2·6 (2·2)		
**Anxiety at 3 months**				
Patient	1·6 (2·0)	1·4 (1·9)	0·39	0·63
Proxy	2·4 (2·2)	2·3 (2·1)		
**Depression at 1 month**				
Patient	1·6 (1·9)	1·5 (1·9)	0·92	0·33
Proxy	2·9 (2·3)	2·7 (2·2)		
**Depression at 3 months**				
Patient	1·5 (1·9)	1·3 (1·8)	0·47	0·11
Proxy	2·7 (2·2)	2·3 (2·2)		
**Independent functioning at 1 month**				
Patient	72·7 (24·3)	73·3 (23·6)	0·87	>0·99
Proxy	37·5 (27·5)	35·3 (26·3)		
**Independent functioning at 3 months**				
Patient	75·4 (24·1)	76·8 (23·2)	0·67	0·35
Proxy	38·7 (27·4)	38·8 (26·6)		
**Health-related quality of life at 1 month**				
Patient	0·5 (0·3)	0·5 (0·3)	0·90	0·37
Proxy	0·2 (0·3)	0·2 (0·3)		
**Health-related quality of life at 3 months**				
Patient	0·5 (0·3)	0·6 (0·3)	0·38	0·30
Proxy	0·3 (0·3)	0·3 (0·3)		
**Overall quality of life at 1 month**				
Patient	6·5 (2·3)	6·4 (2·3)	0·93	0·17
Proxy	4·7 (2·4)	4·9 (2·4)		
**Overall quality of life at 3 months**				
Patient	6·7 (2·2)	6·9 (2·2)	0·36	0·88
Proxy	5·2 (2·5)	5·4 (2·2)		

Data are n (%) or mean (SD).

Details of the sensitivity analyses to address effects of the COVID-19 pandemic

The COVID-19 pandemic had a major effect on hospital admissions and on deaths from March 2020. We therefore did sensitivity analyses (pre-specified after the SAP was published and before the end of the trial) to address effects of the pandemic on the outcomes that were measured over the year post-randomisation. We split the follow-up period into 'before' and 'after' March 1, 2020, for number of emergency readmissions and number of days in hospital, and censored at this date for deaths.

Number of emergency readmissions and time spent in an acute general hospital in the year post-randomisation

We split follow-up into periods of three months anchored by participants' randomisation date. Periods were classed as taking place either before/after March 1, 2020. For any periods that crossed March 1, 2020, we classed them according to whether the majority of days in the period were before or after this date. Accordingly, each participant had four measures of each outcome. We fitted a Poisson model with robust standard errors that allowed for clustering within participant. For number of days in hospital, we used a mixed model with a random effect for participant and allowed different variance estimates by period. These models included treatment allocation, period, hospital and a before/after March 1, 2020 indicator together with all two-way, three-way and four-way interactions between these four factors, together with sex, age and ward. We then estimated hospital-specific treatment effects before and after March 1, 2020, by summing estimates across the four periods. Before and after treatment effects and their interaction were calculated as weighted means of the three hospital-specific treatment effects (with weights proportional to the number of people randomised at each hospital).

Deaths in the year post-randomisation

We estimated treatment effects on deaths before the start of the pandemic, using a Cox proportional hazards model and censoring follow-up on March 1, 2020. We took an analogous approach to the main analysis of this outcome in terms of which covariates to include in the model.

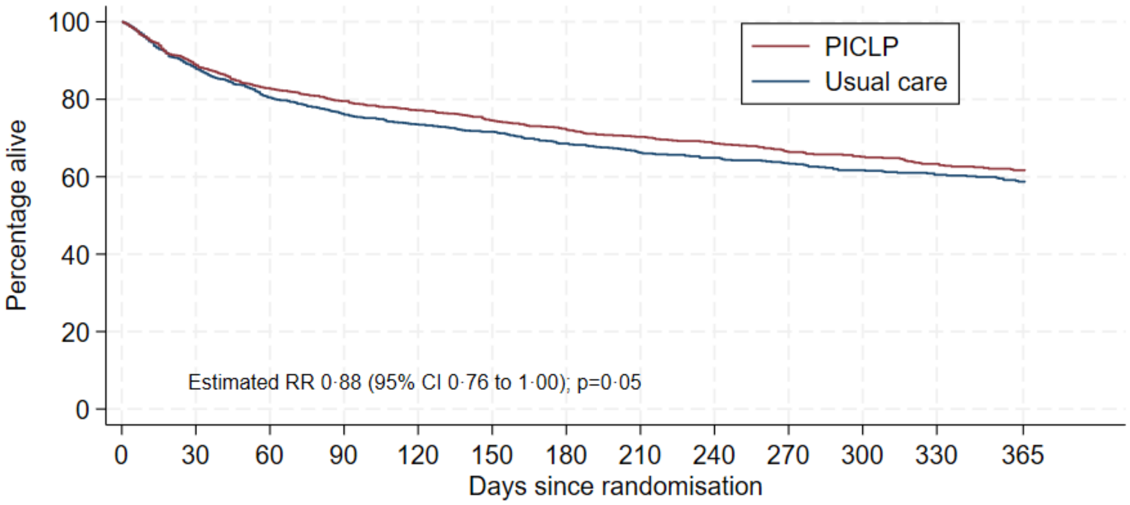

	0	30	60	90	120	150	180	210	240	270	300	330	365
**Usual care**													
At-risk	1364	1153	982	882	798	716	620	552	493	431	382	340	280
Censored	0	55	127	177	230	292	359	407	455	507	544	579	630
Event	0	156	255	305	336	356	385	405	416	426	438	445	454
**PICLP**													
At-risk	1367	1166	1010	926	836	759	668	599	532	460	410	369	304
Censored	0	55	132	176	239	289	359	409	463	519	560	590	646
Event	0	146	225	265	292	319	340	359	372	388	397	408	417

Unit costs

Resource use category	Unit cost	Source
**Clinician time for PICLP delivery**		
**Consultant**	£123 per hour	PSSRU Unit Costs of Health and Social Care, 2021, cost per working hour for a hospital-based consultant psychiatrist
**Assisting clinician**	£52 per hour	PSSRU Unit Costs of Health and Social Care, 2021, cost per working hour for a hospital-based specialist registrar (junior doctor)
**Hospitalisation**		
**Index admission**	£364.35 per day	National Schedule of NHS Costs, 2018/19, cost of a non-elective excess bed day, inflated to 2020/21 costs.
**Readmission**	Varies by HRG currency code	National Schedule of NHS Costs, 2020/21, finished consultant episodes to which HRG groups of clinically similar treatments that consume similar levels of healthcare resource) were assigned to using HRG4+ Reference Costs Grouper software.

Healthcare Resource Group = HRG; PSSRU = Personal Social Services Research Unit

Additional cost-effectiveness data

	Index admission costs	Subsequent admissions costs	PICLP costs	PICLP plus index admission costs	Total costs	Quality-adjusted life years (QALYs)	Life years (LYs)	ICER*	iNHB at λ^* (QALYs) [cost-effectiveness probability]
**Analysis 1 – One-month (30 days) time horizon**									
**Usual care**	£3,985	£1,158	0	£3,985	£5,187	0·0268	0·0781	£77,717, SW	λ_1: 0·0019 [59%]<br>λ_2: 0·0013 [58%]<br>λ_3: 0·0007 [57%]
**PICLP**	£3,769	£1,225	£207	£3,976	£5,152	0·0264	0·0783		
**Difference (95% CI)**	-£216 (-£455 to £23)	£66 (-£223 to £356)	£207 (£200 to £214)	-£9 (-£251 to £232)	-£35 (-£392 to 322)	-0·0004 (-0·0015 to 0·0006)	0·0002 (-0·0010 to 0·0014)		
**Probability PICLP is**	Cost-saving					HRQoL-improving	Life-extending		
	97%	31%	0%	53%	60%	23%	62%		
**Analysis 2 – Three-month (90 days) time horizon**									
**Usual care**	£4,364	£3,750	0	£4,365	£8,143	0·0876	0·2130	£22,191, SW	λ_1: 0·0009 [52%]<br>λ_2: 0·0002 [51%]<br>λ_3: -0·0005 [48%]
**PICLP**	£4,062	£3,898	£207	£4,270	£8,100	0·0857	0·2154		
**Difference (95% CI)**	-£302 (-£618 to £14)	£148 (-£441 to £738)	£207 (£200 to £214)	-£96 (-£414 to £223)	-£42 (-£724 to £640)	-0·0019 (-0·0067 to 0·0029)	0·0023 (-0·0031 to 0·0078)		
**Probability PICLP is**	Cost-saving					HRQoL-improving	Life-extending		
	98%	32%	0%	72%	55%	23%	81%		
**Analysis 3 – Twelve-month (365 days) time horizon****									
**Usual care**	£4,386	£9,475	0	£4,386	£13,921	0·3312	0·7039	Dominated	λ_1: - 0·0132 [38%]<br>λ_2: -0·0112 [36%]<br>λ_3: -0·0092 [35%]
**PICLP**	£4,063	£9,878	£207	£4,270	£14,041	0·3260	0·7225		
**Difference (95% CI)**	-£323 (-£646 to -£0.23)	£403 (-£666 to £1,473)	£207 (£200 to £214)	-£117 (-£442 to £209)	£120 (-£1,036 to £1,277)	-0·0052 (-0·0280 to 0·0176)	0·0186 (-0·0098 to 0·0470)		
**Probability PICLP is**	Cost-saving					HRQoL-improving	Life-extending		
	98%	23%	0%	76%	43%	35%	90%		

All comparisons are PICLP versus usual care. An intervention with an ICER in the southwest quadrant of the cost-effectiveness plane (cost-saving and less effective) is cost-effective if the ICER is above the cost-effectiveness threshold. ICER = incremental cost-effectiveness ratio (incremental cost per QALY), iNHB = incremental net health benefit (includes both health effects on individuals receiving PICLP and health effects on wider NHS patients of additional resources being available or not for other purposes, dependent on the cost implications of PICLP), *compared to PICLP; ** QALYs derived by extrapolation (assumes constant utility after 3 months for those alive beyond that time point), λ = cost-effectiveness threshold, λ_1 £15,000 per QALY; λ_2 , £20,000 per QALY; λ_3, £30,000 per QALY, CI = confidence interval, HRQoL = health-related quality of life, SW = Southwest quadrant.

Please note that disaggregated costs by category were estimated by separate regressions and are therefore indicative and do not add up to the total costs. In each cost category, costs were estimated using generalized linear regression models with an identity link function and a gamma distribution for error terms; these models were adjusted for baseline age, sex and hospital. QALYs were estimated using ordinary least square regression models, and adjusted for baseline EQ-5D-3L, baseline age, sex and hospital. LYs were estimated using ordinary least square regression models, and adjusted for baseline age, sex and hospital. Probabilities were estimated by Monte Carlo simulation. Regression parameter correlations were accounted for using Cholesky decompositions of the variance–covariance matrix for the Monte Carlo simulation.

Data coding and databases

Socioeconomic deprivation

Socioeconomic deprivation was calculated using participants' home addresses (postcodes) and the English Index of Multiple Deprivation (IMD) 2019. The IMD 2019 provides a relative measure of deprivation. It divides England into 32,844 small geographical areas or neighbourhoods and ranks these from the most deprived (ranked 1) to the least deprived (ranked 32,844). The deprivation measure for each geographical area is based on indicators from seven domains – income, employment, health, education, crime, housing and living environment. Participants' postcodes were used to allocate them to the relevant small geographical area. McLennan D, Noble S, Noble M, Plunkett E, Wright G and Gutacker N. The English Indices of Deprivation 2019 Technical Report: Ministry of Housing, Communities and Local Government; 2011. Available at *https://assets.publishing.service.gov.uk/government/uploads/system/uploads/attachment_data/file/833951/IoD2019_Technical_Report.pdf*. Last accessed 9th May 2022.

Rural-urban classification of area of residence

Participants' areas of residence were classified as either rural or urban classification using their home addresses (postcodes) and the English Rural-Urban Classification (RUC) for small area geographies 2011. The RUC classification divides England into 171,372 small areas (Output Areas) using the results of the 2011 Census. These areas are then classified as urban or rural according to their population size. An area with a population size of more than 10,000 people is defined as urban. All other areas are rural.

Bibby P and Brindley P. Urban and Rural Area Definitions for Policy Purposes in England and Wales: Methodology (v1.0). Government Statistical Service, 2013. Available at *https://assets.publishing.service.gov.uk/government/uploads/system/uploads/attachment_data/file/239477/RUC11methodologypaperaug_28_Aug.pdf*. Last accessed 9th May 2022.

Main reason for admission

Data regarding the participant's main reason for admission to hospital were collected from their medical records on the day of recruitment. The recruiter obtained this information from the details recorded by the medical team at the time of admission. The three hospitals where recruitment took place used different medical records systems. The information was obtained from the 'working diagnosis' section of the admission notes for the Oxford site, the 'impression' section of the admission notes for the Exeter site, and the 'reason for attendance' section of the admission notes for the Cambridge site. The notes were transcribed exactly as they had been written by the medical team. The reasons for admission were categorised by three clinicians using consensus. If a participant had multiple documented reasons for admission, we used the first symptom in the list that was specific and definite (i.e. if the first symptom was recorded as '?' or 'possible' and the subsequent was a definite symptom, we used the latter). If a general symptom was followed by a causal one (e.g. weight loss caused by dysphagia) we used the causal symptom.

Medical conditions / multimorbidity

Data regarding the participant's other diagnoses at the time of admission to hospital were collected from their medical records on the day of recruitment. The recruiter obtained this information from the details recorded by the medical team at the time of admission. The three hospitals where recruitment took place used different medical records systems. The information was obtained from the 'significant past medical history' section of the admission notes for the Oxford site, the 'background/past medical history' section of the admission notes for the Exeter site, and the 'hospital diagnoses (problems being addressed during this admission)' and 'non-hospital diagnoses (problems not being addressed during this admission)' section of the admission notes for the Cambridge site. The notes were transcribed exactly as they had been written by the medical team. The medical conditions listed were categorised, by three clinicians using consensus, according to the ICD-10 categories.

We focused on diagnoses that were current, chronic, non-communicable diseases and took an inclusive approach –assuming diagnoses to be current if there was no associated date and including diagnoses recorded as 'recurrent', 'recent', 'probable', 'presumed', 'possible', '??'. We did not include symptoms or signs unless related very obviously to a specific diagnosis, did not translate medications or investigation results into diagnoses, and did not include 'previous' or 'prior' diagnoses unless they were those which were likely to have had permanent effects (e.g. stroke, traumatic brain injury, spina bifida). We assumed that all fractures were recent if the diagnosis was not accompanied by a date, except for fractured neck of femur (trial recruitment did not take place

on any orthopaedic wards meaning that these diagnoses were unlikely to be very recent). We included cancer diagnoses that were accompanied by a date in the last five years or no date was specified.
We did not include infectious diseases or injury and poisoning

Prescribed medications

Participants' prescribed medications were recorded at the time of recruitment and at discharge. These were transcribed by researchers and entered into the study database. Medications were categorised by a clinician. We took an inclusive approach, including all prescriptions that might be considered medications (administered by any route including inhaled and topical) including food supplements, enteral feeds, infusions and total parenteral nutrition. We did not, however, include prescriptions for devices (e.g. inhaler devices, as opposed to inhaled medications), compression stockings, enteral water, saline flushes, dressings. We assumed that anticonvulsant drugs were prescribed for epilepsy (not bipolar disorder), that antihistamine drugs were prescribed for itch (not sedation) and that benzodiazepines were prescribed for anxiety or insomnia except for one prescription of 'as required rectal diazepam' which we assumed to have been prescribed for seizures. We calculated the number and percentage of participants who had been prescribed at least one drug in each of the following categories: antidepressant, antipsychotic, anxiolytic / hypnotic, dementia medications, lithium. We included all relevant drugs whether prescribed at a minimum effective dose or not.

Cognitive function

Cognitive function was measured using the telephone-version of the MoCA (the MoCA-T), which is scored from 0 to 22. We converted the scores to standard MoCA scores (0 to 30) by multiplying MOCA-T scores by 30/22. For the categorisation of participants' baseline scores, we rounded to the nearest whole number then categorised these into severe, moderate, mild and no cognitive impairment.

Databases

We used RRAMP, OpenClinica and Excel databases.

Supplementary post-hoc analyses

Because the primary outcome and several secondary outcomes involved different combinations of discharge, readmission and death over different periods, we did exploratory analyses estimating rates and rate ratios for discharge, readmission and death over time, and estimated the difference in mean total length of stay post-randomisation between groups.

The results show the PICLP group compared with the usual care group to have a slightly increased rate of discharge, starting around 10 days; a slightly increased rate of readmission; and a slightly decreased rate of deaths over the first 150 days. None of these differences were statistically significant, but together they help to illustrate the differing effects of the intervention on the three components of the primary outcome. They demonstrate why the estimated treatment difference for the primary outcome was slightly smaller than for truncated length of index admission, which in turn was smaller than the observed difference between the treatment groups in mean length of index admission. Specifically, neither of these comparisons relating exclusively to the index admission were affected by readmissions or deaths.

Difference in mean total length of stay post-randomisation

	**PICLP (n=1373)**	**Usual care (n=1371)**	**Difference between means**
Mean (SD)	11·19 (11·15)	12·10 (12·58)	-0·84 days<br>95% CI -1·72 to 0·05, p=0·06<br>Bootstrap 95% CI -1.72 to 0.00

Flexible parametric modelling of time to discharge, readmission and death over time

Time to discharge

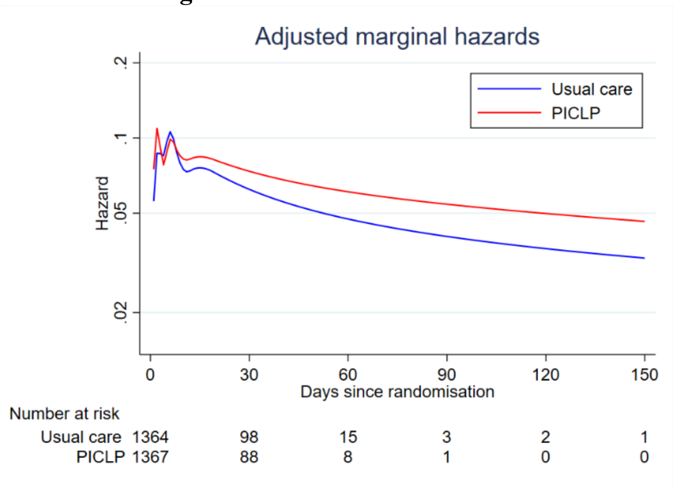

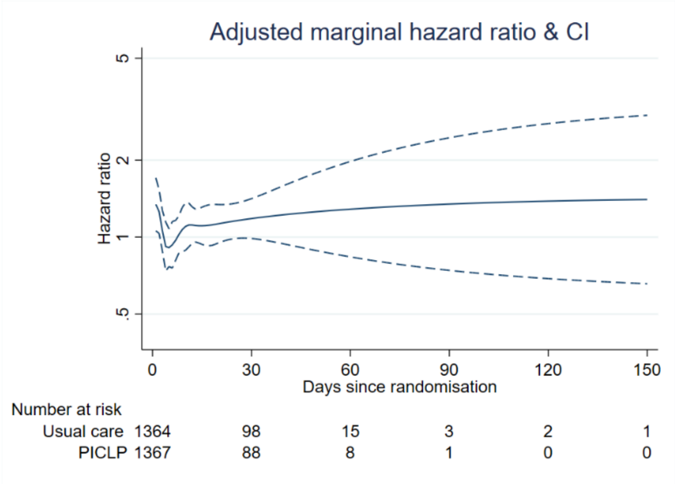

Time to readmission

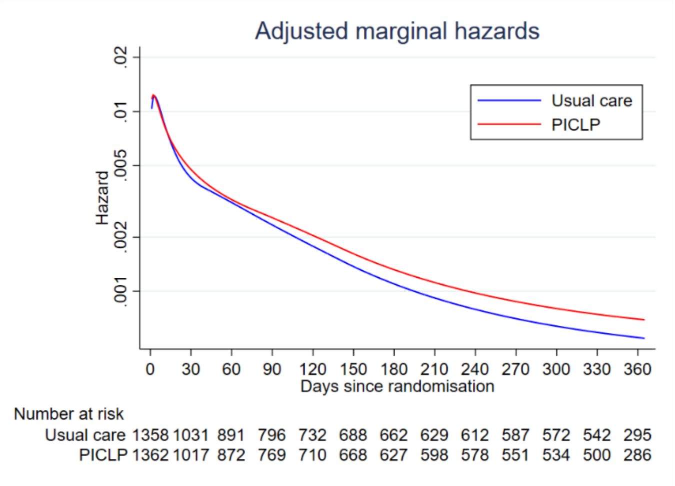

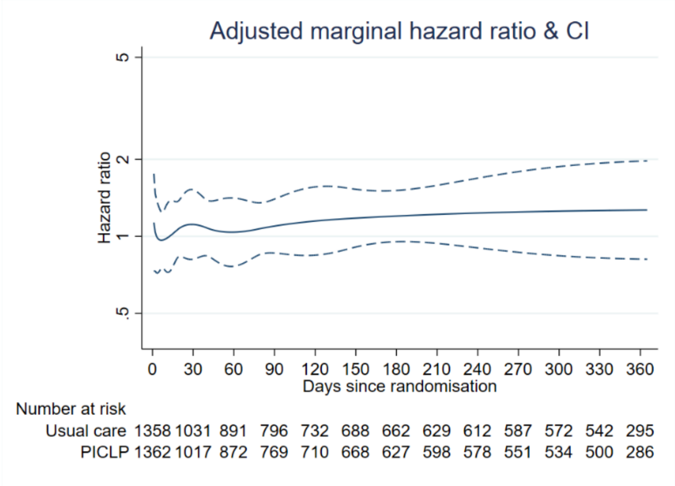

Time to death

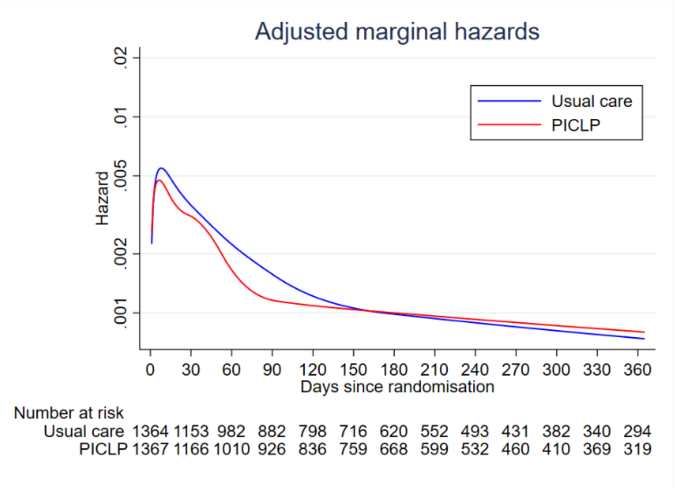

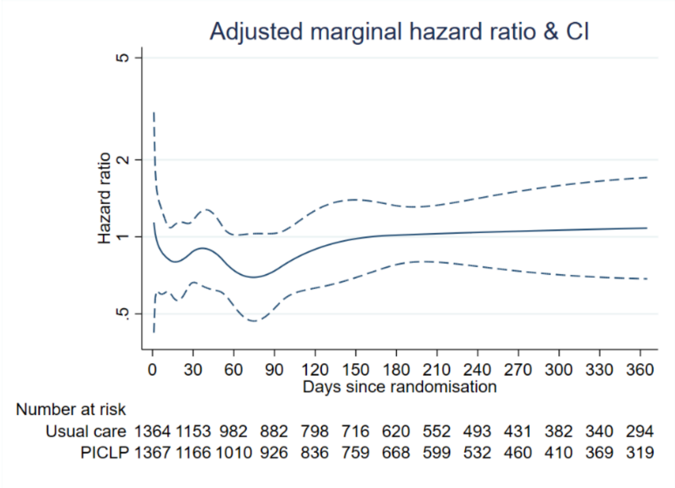

Hazards ratios are PICLP vs usual care.

PROACTIVE INTEGRATED

C0NSULTATION-LIAISON PSYCHIATRY

(PICLP)

SERVICE MANUAL

THE SEVEN PRINCIPLES OF PICLP

1. TAKING A PROACTIVE APPROACH

PICLP is a population-based model. This means that PICLP clinicians proactively assess the psychiatric needs of all patients.

2. PROVIDING CARE PROMPTLY

PICLP clinicians assess patients as soon as possible after admission.

3. MAKING A COMPREHENSIVE BIOPSYCHOSOCIAL ASSESSMENT

The PICLP assessment aims to identify all those problems for which psychiatric intervention might be helpful, using a comprehensive 'biopsychosocial perspective'.

4. FOCUSSING ON A CLEAR GOAL

The main goal of PICLP is to reduce the time that patients spend in hospital. The PICLP clinicians should therefore prioritise the problems that are most likely either to impede the patient's discharge from hospital, or to lead to an early readmission.

5. ENSURING THE SYSTEMATIC DELIVERY OF PSYCHIATRIC CARE

PICLP is systematically delivered according to the manual. A PICLP workbook should be completed for each patient.

6. PROVIDING INTENSIVE INTERVENTIONS

PICLP clinicians work as part of the patient's clinical team to actively deliver the action plan.

7. INTEGRATING WITH THE CLINICAL TEAM

PICLP clinicians are members of the patient's clinical team.

PICLP COMPARED WITH USUAL CONSULTATION-LIAISON PSYCHIATRY

PICLP is population-based rather than referral-based. PICLP is based on the assumption that most medical inpatients benefit from biopsychosocial care and consequently that a population-based service model is most appropriate. This means that PICLP clinicians assess the psychiatric needs of all patients in the relevant wards and do not just react to referrals.

PICLP clinicians assess patients as soon as possible after their admission to hospital. Early involvement allows psychiatric aspects of the patient's care to be prioritised as part of their overall management plan. This contrasts with the later involvement of usual consultation-liaison psychiatry services, whose recommendations are often seen as additions to an already well-established plan.

PICLP takes a biopsychosocial perspective and does not focus solely on psychiatric illness. The assessment seeks to identify all the patient's problems in biomedical, psychological and social domains. It then identifies those problems that could delay the patient's discharge.

PICLP focuses on helping the patient to get home and stay home. This differs from usual consultation-liaison psychiatry which typically focuses on addressing various questions posed by referring clinicians. In PICLP investigations and treatments that do not need to be done in hospital are, where possible, deferred until after discharge. The rationale is that longer hospital stays have a negative effect on older inpatients.

PICLP is delivered systematically according to the manual and workbook, whereas usual consultation-liaison psychiatry interventions are often 'ad hoc'. This systematic approach ensures that problem-based action plans are delivered with consistent quality.

PICLP actions are delivered intensively and assertively by PICLP clinicians, rather than being merely recommended to the medical team, as is usual. They are also continued throughout the patient's stay and adapted with the patient's progress.

PICLP differs from the usual separate consultation-liaison psychiatry model by being fully integrated into the patient's medical care. PICLP clinicians are actively involved in the patient's ongoing care. PICLP also ensures that appropriate discharge plans are in place, and that a patient's 'after discharge care plan' is communicated to relevant out-of-hospital providers.

PICLP CLINICIANS AND ROLES

PICLP is delivered by psychiatrists trained in the psychiatric care of patients with medical illnesses. They are aided in caring for all the patients on the wards they serve by one or more assisting clinicians. These assisting clinicians may be trainee doctors, nurses, occupational therapists, social workers or other appropriately qualified clinicians with experience in the psychiatry of the medically ill.

Psychiatrist

The psychiatrist works collaboratively with the other physicians on the clinical team to ensure a unified perspective is adopted on the patient's problems and their management. This psychiatrist leads the initial biopsychosocial assessment and makes the action plan as there is evidence that initial patient assessments are best done by a senior clinician.

Assisting Clinician

The assisting clinicians extend the psychiatrist's reach. They gather information from records and informants to inform the psychiatrist's assessments. They also deliver the action plans and ensure the daily follow-up of every patient. They liaise with family members and out of hospital services prior to discharge.

Coordination of care

The PICLP clinicians meet at least once each working day to share assessments and plans. Coordination with the patient's other clinicians also occurs daily and includes attendance at ward rounds and multidisciplinary meetings.

PICLP SERVICE DELIVERY

STAGE 1: COMPLETE COMPREHENSIVE BIOPSYCHOSOCIAL ASSESSMENT & PROBLEM LIST

AIM

- To actively seek evidence of ANY current or potential biomedical, psychiatric, psychological or social problems. N.B. the default is to find something.

Think: "what problems does this patient have - in each of the biomedical, psychological and social domains?"

DELIVERED BY

- The PICLP psychiatrist supported by the assisting clinician.

TIMING

- As soon as possible after admission (≤ one working day).

ANTICIPATED TIME REQUIRED

- Average 40 minutes, range 20 to 60 minutes.

ACTIONS

- Always listen and talk to the patient.
- Always review relevant clinical notes.
- Always speak to the patient's doctors, nurses and other healthcare professionals.
- Always review medical treatment especially psychotropic drugs.
- Always find out if the patient is known to community psychiatric services by accessing the relevant records system.
- Consider speaking to primary care team/family/carer/other informants.
  - Identify from the problem list those that are likely to impede discharge or increase risk of early readmission.

OUTCOMES

- Comprehensive and systematic biopsychosocial assessment.
- Problem list covering all of the following areas:
  - ***Bio:*** not medically safe for discharge; medication related problems; sensory deficits.
  - ***Psycho:*** cognitive impairment; behavioural problem(s); substance misuse; other psychiatric diagnoses; other sub-diagnostic threshold psychological problem(s).
  - ***Social:*** functional/mobility impairment(s); social needs/care needs/family concerns; accommodation problem(s); legal considerations.

STAGE 2: CREATE ACTION PLAN

AIM

- To consider the problem list and use it to create an action plan focused on current or potential barriers to prompt discharge.

Think: "Why can't this patient go home today and what do I need to do about it?"

DELIVERED BY

- PICLP clinicians in close collaboration with clinical colleagues.

TIMING

- Soon after biopsychosocial assessment.

ANTICIPATED TIME REQUIRED

- Average 10 minutes, range 5 to 15 minutes.

ACTIONS

- Provide leadership in identifying and managing biopsychosocial problems.
- Create an action plan to address each problem likely to impede discharge.
- Prioritise actions for each problem based on: (a) likelihood the problem will impede discharge; (b) role in driving other problems; (c) how easy it is likely to be to solve.
- Discuss problem list and action plan with the clinical team (plus patient, family/carers, and other hospital staff as indicated).

OUTCOMES

- Action plan focused on addressing problems that are likely to impede discharge and/or increase risk of early readmission.
- Delegation of tasks to specific PICLP clinicians.

STAGE 3: DELIVER ACTION PLAN AND REVIEW DAILY, MODIFYING THE PLAN AS NEEDED

AIM

- To deliver the action plan, monitor progress and adapt the action plan as required with relevant members of the clinical team.

Think: "Why can't this patient still not go home today and what do I need to do about it?"

DELIVERED BY

- The PICLP clinicians in collaboration with other members of the clinical team.

TIMING

- Every weekday
    - Monitor what is preventing the patient from going home that day.
- As required
    - Meet with carer/family members.
    - Liaise with hospital social work/placement team.
    - Liaise with external agencies, social services, and those responsible for the placement.

ANTICIPATED TIME REQUIRED

- Average 10 minutes per working day, range 5 to 30 minutes.

ACTIONS

- Always review the patient's medical records and prescribed medications.
- Always listen and talk to relevant nurses, doctors, social workers and other healthcare professionals.
- When necessary revise the problem list and action plan, including responsibility for actions.
- When necessary listen and talk to the patient.
- When necessary listen and talk to carer/family.
- When necessary advise on prescribed medication.
- When necessary convene team meetings and family meetings.
- When necessary agree actions and establish responsibility for each.
- When changes to discharge plan are needed, discuss promptly with relevant members of the clinical team.
- When necessary arrange biopsychosocial re-assessment.

OUTCOME

- Ongoing identification of barriers to discharge with immediate targeted intervention and delegation of responsibility to specific PICLP clinician.

STAGE 4: ENSURE EFFECTIVE DISCHARGE AND AFTERCARE

AIM

- To ensure the hospital discharge summary includes a specific plan for ongoing management where appropriate.

Think: "What needs to happen out-of-hospital and who needs to know about it?"

DELIVERED BY

- The PICLP clinicians in collaboration with other clinical team members.

TIMING

- Prior to discharge summary completion.

ANTICIPATED TIME REQUIRED

- Average 5 minutes, range 1 to 10 minutes.

ACTIONS

- Liaise with primary care and mental health providers as needed.
- Liaise with other external agencies, social services, and those responsible for the placement.
- Ensure appropriate information in hospital discharge summary.
- Check accuracy of information in hospital discharge summary if included.

OUTCOME

- Appropriate and accurate discharge summary with plans for ongoing management.

TRAINING, SUPERVISION AND QUALITY ASSURANCE

Training

- Training and competency assessments must be completed before clinicians deliver PICLP.

Supervision

- PICLP clinicians will have weekly peer supervision to ensure adherence to the service model and to address problems as they arise.
- Peer supervision will include all hospitals and will be by video-conference.
- Additional face to face meetings will be arranged as needed.

Quality checks

- Quality checks will be undertaken. These will include review of PICLP documentation and direct observation to assess fidelity to the model.

Piloting at each hospital before starting delivery of PICLP

- Deliver PICLP to a minimum of 20 patients from assessment to discharge.
- Pilot for a minimum of two consecutive weeks.
- Each PICLP clinician must be involved in the care of at least 5 patients.

Competency assessments

- Review of 5 fully completed workbooks with assessor using PICLP Quality Control Sheet.
- Observation of one session (half day) of PICLP delivery by assessor using PICLP Quality Control Sheet.
- Psychiatrists assessed on delivering biopsychosocial assessment and daily follow up.
- Assistants assessed on daily follow up.

DO’S AND DONT’S FOR PICLP CLINICIANS

DO

- Remember that you are delivering a new model of care, not your old one
- Be proactive in both the patient’s assessment and treatment
- Elicit problems in all biomedical, psychological and social domains – not just one of these
- Identify problems that may impede prompt discharge or lead to early readmission
- Provide robust intensive interventions for these problems
- Remember that problems and targets of intervention may evolve over time
- Work closely with the medical team, nurses and associated health professionals
- Keep on going - actively caring for the patient from admission to discharge

DON’T

- Revert to your usual consultation-liaison psychiatric practice
- Jump on one problem without considering the whole list
- Only consider psychiatric illness
- Forget to talk to nurses and relatives
- Let your input dwindle over the duration of the admission
- Act without close collaboration with other staff caring for the patient
- Try to treat problems in hospital that could be treated post-discharge
- Focus on problems which cannot affect the discharge date

HOW TO RESPOND TO FREQUENTLY ASKED QUESTIONS

Who are you?
I am a doctor/clinician working on this ward. I work with the rest of your medical team to make sure we address all your problems, not just the medical ones.

What is PICLP?
It is a new way of delivering care by having psychiatrists work as part of the medical team. It means that we can better address a wide range of problems and help you leave hospital as soon as you are ready to.

Why is it bad to stay in hospital?
Coming into hospital can be really helpful when people have serious medical problems that need to be assessed and treated. But staying in hospital for longer than necessary can have bad effects – especially for older people. People can lose their confidence, independence and mobility if they stay in hospital too long. They can also get hospital acquired infections. So it's important that people are in hospital only long enough to get on top of their medical problems and not so long that they suffer bad effects from being here.

I don't have a psychiatric problem – why are you seeing me/my relative?
We work to help with a wide range of problems, not just psychiatric illness. We do that by working with the rest of the team to make sure we're addressing all the problems that may delay you going home, not just medical ones.

PROBLEMS, IMPEDIMENTS TO DISCHARGE AND ACTION PLANS: CATEGORIES AND EXAMPLES

	Problem category	Category description	Example		
			**Problem**	**Impediment to discharge**	**Action plan**
Bio-	Not medically safe for discharge	Active medical conditions (acute or chronic) requiring urgent treatment in the general hospital.	Patient has severe pneumonia.	Need for intravenous antibiotics.	Check with the medical team how soon they can start to give the antibiotics orally and plan for discharge that day.
	Medication-related problems	Non-concordance, polypharmacy, side-effects, physician concerns.<br>*N.B. Not restricted to psycho-active medications*	Patient admitted after a fall. Usual antidepressant prescription stopped by medical team.	• Patient has become severely anxious and is reluctant to mobilise in case they fall again.	Discuss risks and benefits of restarting antidepressant medication with medical team. Restart or switch to a different drug to reduce anxiety, allowing mobilisation and prompt discharge.
	Sensory deficits	Sensory impairment that affects the patient's orientation, ability to engage with care or interactions with staff.	Patient's hearing aids and spectacles have been left at home.	Patient's cognitive function and need for care after discharge are both difficult for the ward team to assess.	Work with the ward team to resolve impairments (e.g. find hearing aid) and assist with cognitive testing to ensure best discharge destination can be decided promptly.

	Problem category	Category description	Example		
			Problem	Impediment to discharge	Action plan
Psycho-	Cognitive impairment	Acute or chronic cognitive impairment.	Patient has delirium and possible dementia.	Patient is suspicious of nursing staff and refuses medications.	Work with medical team to treat delirium. Gather information from family to assess usual cognitive function. Ensure an appropriate early discharge plan.
	Behavioural problems	Any patient behaviour that affects their care.	Patient with dementia shouts at staff telling them to leave him alone.	• Ward staff are unable to assess patient's symptoms and are avoiding the patient.	Educate the ward staff about behaviours in dementia.<br>Advise on engaging the patient in care so that symptoms can be assessed and treated promptly.
	Substance misuse	Misuse of any substance.	Patient has smoked cigarettes for 20 years, does not want to stop.	Patient is agitated on ward and unable to engage with rehabilitation.	Advise on nicotine replacement to allow rehabilitation. Ensure the planned post-discharge destination allows smoking.
	Psychiatric diagnoses	Psychiatric conditions other than delirium and dementia.	Patient has depression and is negative about discharge home.	Staff avoid spending time with patient, leading to slow discharge planning.	Advise on antidepressant medication, educate ward staff about depression and provide behavioural activation to treat depression and engage patient in planning for early discharge.
	Sub-diagnostic threshold psychological problems	Psychiatric or psychological problems that do not meet diagnostic criteria.	Patient is anxious about being in cramped places.	Patient is reluctant to have a scan to investigate abdominal pain; scan is repeatedly deferred, delaying discharge.	Discuss pros and cons of scan, and other options, with patient and medical team to ensure a decision and prevent further delays to discharge.

	Problem category	Category description	Example		
			Problem	Impediment to discharge	Action plan
social	Problems with basic activities of daily living	Dependence on the assistance of others (prior to admission or currently) for basic needs.	Patient's mobility has declined, now unable to walk to bathroom alone.	Unclear whether patient will manage activities of daily living without assistance post-discharge.	Work with the ward team to determine causes of the decline and the need for carers or new living arrangements to plan prompt discharge.
	Problems with instrumental activities of daily living	Inability to do activities that maintain independence and quality of life.	Patient has had a stroke, can no longer drive to local shops.	• Patient cannot get food shopping, does not want to move to care home.	Work with patient, family, occupational therapist and social worker to find best care solution, avoiding discharge delay.
	Accommodation problems	Isolated or inappropriate accommodation, tenancy or residency problems.	Patient lives in residential home, but the home is unwilling to take them back due to increased care needs.	Patient potentially has no accommodation to return to.	Check residential home's understanding of care needs is accurate. Work with patient, family and ward team to arrange return to that home or an alternative.
	Legal problems	Patient's capacity to make treatment or discharge decisions is uncertain.<br>Problems regarding power of attorney or advance directive.	Patient with mild dementia is unwilling to accept additional home care post-discharge. The ward team is concerned they will forget to take essential medications.	There are differing opinions in the ward team about whether the patient should be allowed to go home without care, leading to a delay in discharge planning.	Assess the patient's capacity to make discharge decisions. Educate the ward team about capacity. Discuss wishes with patient and arrange a 'best interests' meeting if required, to avoid further delays to discharge.

Proactive Integrated

Consultation-Liaison Psychiatry (PICLP)

Workbook

Patient initials		Age		Sex (M/F)	
Ward		Bed		Medical team	

The Seven PICLP Principles	
1	**TAKING A PROACTIVE APPROACH**
2	**PROVIDING CARE PROMPTLY**
3	**MAKING A COMPREHENSIVE BIOPSYCHOSOCIAL ASSESSMENT**
4	**FOCUSSING ON A CLEAR GOAL**
5	**ENSURING THE SYSTEMATIC DELIVERY OF PSYCHIATRIC CARE**
6	**PROVIDING INTENSIVE INTERVENTIONS**
7	**INTEGRATING WITH THE CLINICAL TEAM**

**Patient identifiers**										
First name										
Last name										
Date of birth	D	D	M	M	Y	Y				
Hospital number										

**Admission details**							
Date of admission	D	D	M	M	Y	Y	

**Pre- face to face assessment information**			
Reason for admission			
Psychiatric diagnoses on admission		Currently under mental health service care<br>Y or N	
Mobility, social support, carers and accommodation on admission			

**Pre- face to face assessment background notes**

Stage 1: Complete biopsychosocial assessment and problem list

Sources of information reviewed: Y or N or U (unavailable)				Discussion with: Y or N			
Hospital notes		Mental health team notes		Patient		Clinical (ward) team	
Drug chart		Primary care notes		Family/Carer		Other professionals	

	Categories to consider for potential problems	Present Y or N	If problem present, please give details	Is problem impeding discharge or increasing risk of readmission? Y or N
Bio -	Not medically safe for discharge			
	Medication-related problems			
	Sensory deficits			
Psycho -	Cognitive impairment			
	Behavioural problems			
	Substance misuse			
	Psychiatric diagnoses			
	Sub-diagnostic threshold psychological problems			
Social	Problems with basic activities of daily living			
	Problems with instrumental activities of daily living			
	Accommodation problems			
	Legal problems			

Date Stage 1 completed	D	D	M	M	Y	Y
Time taken to complete Stage 1 only (minutes to nearest 5)	Psychiatrist					
	Assisting Clinician					

Stage 2: Create action plan for discharge

The plan has been discussed with: Y or N

Patient		Medical team		Therapy (OT/PT) team		Discharge/Social Work team	
Family/Carer		Nursing team		Other (detail):			

** Who is responsible for this action point – Psychiatrist (S), Assisting Clinician (A)

	Categories	**Action plan:** for PICLP clinicians to address problems likely to impede discharge or increase risk of readmission	S or A**
Bio -	Not medically safe for discharge		
	Medication-related problems		
	Sensory deficits		
Psycho -	Cognitive impairment		
	Behavioural problems		
	Substance misuse		
	Psychiatric diagnoses		
	Sub-diagnostic threshold psychological problems		
Social	Problems with basic activities of daily living		
	Problems with instrumental activities of daily living		
	Accommodation problems		
	Legal problems		

Date Stage 2 completed	D	D	M	M	Y	Y
Time taken to complete Stage 2 only (minutes to nearest 5)	Psychiatrist					
	Assisting Clinician					

Stage 3: Deliver action plan and review daily, modifying plan as needed

* Days since randomisation		Barriers to discharge				PICLP clinician actions		
Day*	Date<br>DD/MM/YY	Bio	Psycho	Social	WHY can't the patient be discharged TODAY?	** Psychiatrist (S), Assisting Clinician (A)<br>What ACTIONS for PICLP clinicians today?	S or A**	Time (mins) nearest 5 min
		Impeding discharge: Y or N						
0							S	
							A	
1							S	
							A	
2							S	
							A	
3							S	
							A	
4							S	
							A	
5							S	
							A	
6							S	
							A	
7					Biopsychosocial re-assessment completed? Y or N, if N explain			
							S	
							A	

Stage 3: Deliver action plan and review daily, modifying plan as needed									
* Days since randomisation		Barriers to discharge				PICLP actions			
Day*	Date<br>DD/MM/YY	Bio	Psycho	Social	WHY can't the patient be discharged TODAY?	**Psychiatrist (S), Assisting Clinician (A)<br>What ACTIONS for PICLP clinicians TODAY?		S or A**	Time (mins) nearest 5 min
		Impeding discharge: Y or N							
8								S	
								A	
9								S	
								A	
10								S	
								A	
11								S	
								A	
12								S	
								A	
13								S	
								A	
14					Biopsychosocial re-assessment completed? Y or N, if N explain				
								S	
								A	

Stage 3: Deliver action plan and review daily, modifying plan as needed

* Days since randomisation		Barriers to discharge				PICLP clinician actions		
Day*	Date	Bio	Psycho	Social	WHY can't the patient be discharged TODAY?	**Psychiatrist (S), Assisting Clinician (A)<br>What ACTIONS for PICLP clinicians TODAY?	S or A**	Time (mins) nearest 5 min
	DD/MM/YY	Impeding discharge: Y or N						
15							S	
							A	
16							S	
							A	
17							S	
							A	
18							S	
							A	
19							S	
							A	
20							S	
							A	
21					Biopsychosocial re-assessment completed? Y or N, if N explain			
							S	
							A	

**Stage 3: Deliver action plan and review daily, modifying plan as needed**									
* Days since randomisation		Barriers to discharge				PICLP clinician actions			
Day*	Date<br>DD/MM/YY	Bio	Psycho	Social	WHY can't the patient be discharged TODAY?	**Psychiatrist (S), Assisting Clinician (A))<br>What ACTIONS for PICLP clinicians TODAY?		S or A**	Time (mins) nearest 5 min
		Impeding discharge: Y or N							
22								S	
								A	
23								S	
								A	
24								S	
								A	
25								S	
								A	
26								S	
								A	
27								S	
								A	
28					Biopsychosocial re-assessment completed? Y or N, if N explain				
								S	
								A	

Stage 3: Deliver action plan and review daily, modifying plan as needed

* Days since randomisation		Barriers to discharge				PICLP clinician actions		
Day*	Date	Bio	Psycho	Social	WHY can't the patient be discharged TODAY?	**Psychiatrist (S), Assisting Clinician (A)<br>What ACTIONS for PICLP clinicians TODAY?	S or A**	Time (mins) nearest 5 min
	DD/MM/YY	Impeding discharge: Y or N						
29							S	
							A	
30							S	
							A	

Stage 4: Discharge

Date PICLP ended	D	D	M	M	Y	Y

Psychiatric diagnoses during admission (all) including ICD-10 code	
Inform post-discharge Primary Care Physician of assessment in hospital discharge summary Y or N	
Discharge Plan (Detail)	

Time taken to complete Stage 4 only (minutes to nearest 5)		
	Psychiatrist	
	Assisting Clinician	
